# Gut Microbiota-Derived Diaminopimelic Acid Promotes the NOD1/RIP2 Signaling Pathway and Plays a Key Role in the Progression of Severe Acute Pancreatitis

**DOI:** 10.3389/fcimb.2022.838340

**Published:** 2022-06-22

**Authors:** Juying Jiao, Jianjun Liu, Qi Li, Guixin Zhang, Chen Pan, Fei Luo, Qingkai Zhang, Bing Qi, Liang Zhao, Peiyuan Yin, Dong Shang

**Affiliations:** ^1^ Clinical Laboratory of Integrative Medicine, The First Affiliated Hospital of Dalian Medical University, Dalian, China; ^2^ College of Integrative Medicine, Dalian Medical University, Dalian, China; ^3^ Shanghai Medical College, Fudan University, Shanghai, China; ^4^ Key Laboratory of Separation Science for Analytical Chemistry, Dalian Institute of Chemical Physics, Chinese Academy of Sciences, Dalian, China; ^5^ Pancreaticobiliary Centre, Department of General Surgery, The First Affiliated Hospital of Dalian Medical University, Dalian, China

**Keywords:** gut microbiota, severe acute pancreatitis, NOD1 signaling pathway, diaminopimelic acid (DAP), Qingyi Keli (QYKL)

## Abstract

Impaired intestinal barrier function and gut microbiota dysbiosis are believed to be related to exacerbation of acute pancreatitis (AP). As a bacterial cell wall peptidoglycan component, diaminopimelic acid (DAP) is a specific ligand of NOD1 that regulates the NOD1/RIP2/NF-kB signaling pathway. Here, we investigated the role of DAP in the crosstalk between the gut microbiota and pancreas during the occurrence of AP. Upregulation of NOD1/RIP2/NF-kB and elevated serum DAP levels were found in severe AP (SAP) model rats. The accumulation of DAP in SAP patients corroborated its ability to serve as an indicator of disease severity. Subsequently, SAP rats were treated with oral administration of the traditional Chinese medicine Qingyi Keli (QYKL) as well as neomycin, which can widely eliminate DAP-containing bacteria. Both QYKL and neomycin intervention ameliorated intestinal and pancreatic damage and systemic inflammation in SAP rats. Through 16S rDNA sequencing, we found that QYKL could rehabilitate the gut microbiota structure and selectively inhibit the overgrowth of enteric bacteria, such as Helicobacter and Lactobacillus, in SAP rats without affecting some protective strains, including Romboutsia and Allobaculum. Interestingly, we demonstrated that the decrease in serum DAP was accompanied by suppression of the NOD1/RIP2/NF-kB signaling pathway in both the intestine and pancreas of the two intervention groups. Taken together, these results suggested that the gut microbiota-DAP-NOD1/RIP2 signaling pathway might play a critical role in the progression of AP and that SAP could be alleviated *via* intervention in the signaling pathway. Our work provides new potential early warning indicators of SAP and targets for intervention.

## 1 Introduction

Severe acute pancreatitis (SAP) accounts for 15%-20% of acute pancreatitis (AP), and the mortality is up to 30% (Forsmark et al., 2016). Infection of necrotic pancreatic tissue is one of the main causes of high mortality (Frossard et al., 2008). The intestine is an organ that is in close proximity to the pancreas and is damaged during the early stage of SAP because of its sensitivity to ischemia-hypoxia, leading to increased permeability and disturbed microbiota structure (Cen et al., 2018). Consequently, bacterial translocation from the incomplete intestinal barrier will induce complications such as pancreatic tissue infection (Signoretti et al., 2017). Studies have shown that the gut microbiota structure varies with the progression of SAP and is closely related to disease severity (Tan et al., 2015; Zhu et al., 2018). Aberrant bacteria or their metabolites entering the blood circulation through the injured gut barrier prompt disease aggravation toward systemic inflammation and multiple organ failure, which would further destroy the gut microbiota and lead to a vicious cycle that is difficult to reverse (Capurso et al., 2012; Watanabe et al., 2017). Therefore, it is of great significance to conduct a thorough study into the interplay between microbiota and host, which may shed light on the mechanism of AP progression.

Abnormal activation of trypsinogen within pancreatic acinar cells was acknowledged to initiate pancreatic injury, while persistent pancreatic inflammation was reported to be sustained partly by pathogen-associated molecular patterns (PAMPs) from translocated bacteria (Watanabe et al., 2017). PAMPs can bind to host pattern recognition receptors such as Toll-like receptors (TLRs) and nucleotide oligomerization and binding domain (NOD)-like receptors (NLRs) to activate inflammatory signaling and induce cytokine production, which in turn induces a cascade of inflammatory reactions, leading to systemic inflammatory response syndrome (SIRS) and multiple organ dysfunction syndrome (MODS). Studies focusing on TLRs, such as the interaction between TLR4 and lipopolysaccharide (LPS), have established a well-known link between the microbiota and the host (Sharif et al., 2009; Cao et al., 2018). Recently, other cytosolic receptor NLRs have attracted increasing attention for their significant role in inflammation and immunity, especially in SAP (Clarke et al., 2010; Xu et al., 2018). Tsuji et al. showed that NOD1 activation in acinar cells by gut microbiota played an indispensable role in the occurrence of cerulein-induced AP (Tsuji et al., 2012), where diaminopimelic acid (DAP) from cell wall peptidoglycans of gram-negative bacteria and particular gram-positive bacteria is a ligand for NOD1 that can induce innate immune responses. Once DAP is recognized by NOD1, which is widely expressed in the intestine, pancreas and most immune cells, it can activate receptor-interacting protein 2 (RIP2) and downstream signaling pathways, including NF-κB, JNK, and MAPK, to produce a large amount of inflammatory cytokines (Girardin et al., 2003; Caruso et al., 2014; Canas et al., 2018). Accordingly, we hypothesized that the activation of the NOD1 signaling pathway by DAP might affect the pathogenesis and development of AP.

Recovering gut microecology balance and maintaining the integrity of the intestinal barrier are favorable ways to restrain inflammation and relieve extensive lesions in the treatment of AP in practice. Qingyi decoction has been proven to be effective *via* multiple pharmacological mechanisms and to have fewer adverse effects for AP patients in the clinic for decades (Chen et al., 2015; Zhang et al., 2015; Su et al., 2019; Zhang et al., 2019). Qingyi Keli (QYKL; hospital preparations of the First Affiliated Hospital of Dalian Medical University), as a granular formulation modified from Qingyi decoction, has greatly improved the medication compliance of patients. The main components of QYKL consist of Radix bupleur, Scutellaria baicalensis, Gardenia, Radices paeoniae alba, Corydalis tuber, Radices saussureae, Rheum officinale and Glauber’s salt. It has been reported that Qingyi decoction could promote blood circulation, reduce inflammation, prevent oxidative damage and protect intestinal barrier function (Yang et al., 2009; Zhang et al., 2015; Xiang et al., 2017). However, oral administration as the traditional route for Chinese herbs implies that there are likely interactions between the drugs and the enteral microbiota that contribute to the effects of Qingyi decoction, which has not been explored thus far. In addition, antibiotics are indispensable for infection control. Neomycin, as a gut-restricted aminoglycoside antibiotic, can inhibit DAP-containing bacteria (Hergott et al., 2016). As it is rarely absorbed after oral administration, neomycin plays a role mainly through its impact on the gut microflora. Therefore, the aim of this study was to evaluate the effect of the gut microbiota-DAP-NOD1/RIP2 signaling axis on the progression of AP as well as the intervention mechanism of QYKL and neomycin.

In this study, we utilized 16S rRNA gene sequencing to profile the gut microbiota structure of SAP rats and observed the regulatory effects of QYKL and neomycin. In addition, UHPLC-TQMS (triple quadrupole mass spectrometry) was employed to establish a derivative method for quantitative analysis of DAP in the serum of SAP rats, which could provide direct evidence of the entry of DAP into the circulatory system. Subsequently, the changes in DAP levels were verified by serum samples from patients with AP of different severities. Finally, animal experiments were conducted to explore whether intervention with QYKL and neomycin could ameliorate SAP by regulating the gut microbiota-pancreatic axis. This work has clinical significance, as it demonstrates that the gut microbiota-DAP-NOD1/RIP2 signaling axis participates in the pathological process of SAP and provides new potential targets for early warning and intervention of SAP.

## 2 Materials and Methods

### 2.1 Experimental Animals

Male SPF Sprague–Dawley rats weighing 200-220 g were provided by the Experimental Animal Center of Dalian Medical University and were maintained in a temperature-controlled room with free access to food and water. Animal operations conformed to the guidelines for the Care and Use of Laboratory Animals of the National Institutes of Health. All protocols were approved by the Ethics Committee of Animal Experiments of Dalian Medical University.

### 2.2 Clinical Samples

Serum samples from 153 subjects including 97 AP patients and 56 healthy adults were collected from the Department of Abdominal Emergency Surgery and Physical Examination Center of the First Affiliated Hospital of Dalian Medical University. Patients meeting the diagnostic criteria of AP were stratified into three groups by clinical severity, that is 68 mild AP (MAP), 13 moderately severe AP (MSAP), and 16 severe AP (SAP) according to the revised Atlanta classification criteria (Banks et al., 2013). Patients with gastrointestinal diseases, tumors and severe chronic underlying diseases were excluded, and those with a time from the onset of AP symptoms to sampling exceeding 48 h or transferred from other hospitals were also excluded. The serum samples of AP patients were collected upon admission. Healthy subjects (HCs) were recruited who were matched with AP patients for age, sex and body mass index (BMI) and were free of chronic metabolic, gastrointestinal, cardio-cerebrovascular, and neoplastic diseases. The demographic and clinic characteristics of all participants can be found in supplementary **Table S1**. The associated trial protocols were approved by the Ethics Committee of the First Affiliated Hospital of Dalian Medical University (YJ-KS-KY-2019-93), and informed consent was obtained from all participants.

### 2.3 SAP Model Establishment, Grouping and Sample Collection

SAP models were established according to previous reports (Huang et al., 2017). Briefly, rats under anesthesia were administered a standard biliopancreatic duct retrograde infusion of freshly prepared 3.5% sodium taurocholate (Sigma–Aldrich, USA) at a dosage of 0.1 ml/100 g body weight. Rats in the sham operation (SO) group were given a sham surgery in which the pancreas and intestine were flipped following laparotomy. Sixty rats were randomly assigned into four groups: the SO group (n=12), severe acute pancreatitis group (SAP, n=20), Qingyi Keli treatment group (QYKL, n=16) and neomycin pretreatment group (Neo, n=12). Rats in the QYKL group were intragastrically given the hospital preparation “Qingyi Keli” (The First Affiliated Hospital of Dalian Medical University) at a dosage of 0.18 g/ml and 10 ml/kg body weight 2 h and 12 h after the operation. Rats in the SO and SAP groups were given an equivalent dosage of water. Rats in the Neo group were pretreated with neomycin (1 g/L, Solarbio Life Sciences) in drinking water for 3-4 weeks before modeling. Neomycin, an aminosaccharide antibiotic, is a DAP-containing bacteria-sensitive antibiotic. It was used as a positive control for comparison with the Qingyi Keli group.

Rats were sacrificed 24 h after modeling, and blood samples were collected from the aorta abdominalis, centrifuged to obtain plasma, aliquoted and stored at -80°C. The pancreas and distal ileum were removed and fixed in 4% paraformaldehyde for histopathological observation, and another portion was snap-frozen with liquid nitrogen and then stored at -80°C for western blotting and RT–PCR.

### 2.4 Histopathology

Formaldehyde-fixed pancreas and intestine tissues were embedded in paraffin and then sectioned into 4-μm slices. Afterward, tissue samples were stained with hematoxylin-eosin (HE) and observed under a light microscope. Histological scoring was evaluated according to Rongione’s standard (Rongione et al., 1997).

### 2.5 Measurements of Plasma Amylase, Lipase, Cytokines and Diamine Oxidase (DAO)

The concentrations of amylase, lipase, TNF-α, IL-1β, IL-6, procalcitonin (PCT) and diamine oxidase (DAO) in plasma were measured by commercially available enzyme-linked immunosorbent assay (ELISA) kits according to the manufacturer’s instructions (Mlbio, Shanghai, China).

### 2.6 DNA Extraction From Colonic Contents

Total bacterial DNA from colonic contents in each group was extracted according to the manufacturer’s instructions of the QIAamp DNA stool mini kit (Qiagen, Germany). The DNA concentration and purity were measured spectrophotometrically using simpliNano (biochrom, UK). According to the concentration, DNA was diluted to 1 ng/μL using sterile water.

### 2.7 Library Preparation, Sequencing and Analysis

The V3–V4 region of bacterial 16S rDNA was amplified using specific primers with barcodes. The PCRs were carried out in 30-μL reactions with 10 ng of template DNA, 0.2 μM of each primer, and 15 μL of Phusion^®^ High-Fidelity PCR Master Mix with GC buffer (New England Biolabs). The reaction procedure was as follows: initial denaturation at 98°C for 1 min; followed by 30 cycles of denaturation at 98°C for 10 s, annealing at 50°C for 30 s, and elongation at 72°C for 30 s; and finally, 72°C for 5 min. PCR products were detected by 2% agarose gel and were purified with the GeneJET Gel Extraction Kit (Thermo Fisher Scientific, USA). Sequencing libraries were generated using the Ion Plus Fragment Library Kit 48 rxns (Thermo Fisher Scientific, USA) following the manufacturer’s instructions. The library quality was assessed on the Qubit@ 2.0 Fluorometer (Thermo Fisher Scientific, USA) and Agilent Bioanalyzer 2100 system. Finally, the library was sequenced on the IonS5™XL platform (Thermo Fisher Scientific, USA).

The obtained raw reads were filtered by QIIME quality filters to obtain the effective data (clean reads). Then, we used pick_de_novo_otus.py to pick operational taxonomic units (OTUs) by making an OTU table. Sequences with ≥97% similarity were assigned to the same OTUs. We picked a representative sequence for each OTU and used the Ribosomal Database Project (RDP) classifier to annotate taxonomic information for each representative sequence (Wang et al., 2007). Principal coordinate analysis (PCoA) of core OTUs was performed using the weighted gene coexpression network analysis (WGCNA), stats and ggplot2 packages of R software, and community diversity was measured by the Shannon–Weiner biodiversity index (Shannon index). This work was conducted by Novogene Institute (Beijing, China).

### 2.8 Expression of Intestinal Barrier Proteins and the NOD1 Signaling Pathway

#### 2.8.1 Western Blotting

Expression of intestinal barrier proteins and the NOD1 signaling pathway in the intestine and pancreas was evaluated by western blotting. First, tissue samples were homogenized in lysis buffer (KeyGEN BioTECH, China) to obtain total proteins. Second, protein concentrations were determined using the BCA Protein Assay Kit according to the product manual (KeyGEN BioTECH, China). Third, proteins were denatured and then separated by 8% SDS–PAGE and electroblotted onto polyvinylidene fluoride membranes. Fourth, membranes were blocked with 5% skimmed milk for 2 h and then incubated with primary antibody at a dilution of 1:500 for occludin and claudin 2; 1:1000 for NOD1 (CST, USA), RIP2 (Abcam, USA), phospho-NF-κB p65 (Abclonal, China) and NF-κB p65 (Abcam, USA); and 1:8000 for β-actin (Abcam, USA) in blocking buffer at 4°C overnight with gentle shaking. Fifth, after washing with Tris-buffered saline Tween-20 to remove excess primary antibody, the membranes were incubated with HRP-conjugated secondary antibody (Abcam, USA) at a dilution of 1:5000 in blocking buffer for 1 h at room temperature and then washed again. Finally, bands were detected by chemiluminescence using a gel imaging system (Tanon 5200, China).

#### 2.8.2 RT–qPCR

The RNA levels of members of the NOD1 signaling pathway were detected by RT–qPCR in the intestine and pancreas using PrimeScript RT and SYBR Premix Ex TaqTM II kits (Takara, Japan) according to the product manual. Briefly, tissue RNA was extracted followed by reverse transcription into cDNA, and then PCR was performed with cDNA as the template. Primers for PCR included the following: NOD1 5’- GCCCGACAGAAACTCCTTCA-3’ and 5’-GGCATGGCATGTACCTGGTTA-3’; RIP2 5’-AGAGGGAAGCCATTGTGAGC-3’ and 5’-GGTCCTTGTAGGTTTGGTGC-3’; NF-κB p65 5’-CGTGAGGCTGTTTGGTTTG-3’ and 5’-CTGTCTTATGGCTGAGGTCTG-3’; and β-actin 5’-GGAGATTACTGCCCTGGCTCCTA-3’ and 5’-GACTCATCGTACTCCTGCTTGCTG-3’. The PCR procedure involved two-stage standard amplification, including 1 cycle of predenaturation at 95°C for 30 s and 40 cycles of denaturation, annealing and extension at 95°C for 5 s and 60°C for 34 s. The cycle threshold (CT) values were used to calculate the relative mRNA expression through the 2^-ΔΔCT^ method.

### 2.9 Establishment of a Mass Spectrometry Detection Method for Measuring Serum DAP Levels Based on UHPLC-TQMS

Derivatization was employed to establish a method for the detection of serum DAP by the UHPLC-TQMS platform, including serum sample pretreatment, 6-aminoquinolyl-N-hydrosysuccinimidyl carbamate (AQC) derivatization, chromatographic separation and mass spectrometry detection.

#### 2.9.1 Sample Preparation and Derivatization

One hundred microliters of each plasma sample was added to 400 µL of methanol containing Lys-d4 (1 ng/mL) as an internal standard. Next, the sample was vortexed for 30 s to extract DAP and centrifuged at 20,000 g for 10 min (10°C). Then, 400 µL of supernatant was collected to freeze-dry. An AccQ Tag™ Ultra Derivatization Kit (Waters, USA) was utilized for derivatization according to the instructions. Briefly, 70 µL of borate buffer and 20 µL of AQC were added to freeze-dried samples. The sample was vortexed for 30 s. Then, the tube was sealed and incubated at 55°C for 10 min. After the reaction, the sample was cooled on ice for 1 min.

#### 2.9.2 Chromatographic Separation and Mass Spectrometry Detection

UHPLC-TQMS analysis was performed using a Waters Xevo TQ-XS triple quadrupole mass spectrometer (Waters, US). An ACQUITY UPLC C8 column (50 × 2.1 mm, 1.7 µm) was used for separation. The column temperature was 50°C. The sample room temperature was 10°C. The flow rate was 0.3 mL/min. Phase A was 0.1% formic acid/Milli-Q water. Phase B was 0.1% formic acid/acetonitrile. Gradient elution for sequence analysis was performed as follows: 0 min, 2% B; 5 min, 9.1% B; 6 min, 10% B; 8~9 min, 95% B; 10~11 min, 2% B. Multiple reaction monitoring (MRM) mode was used to quantify DAP and Lys-d4. The precursor ion and daughter ion were 266.1 > 171.1 and 321.2 > 171.1, respectively. The collision energy was 10 eV for both compounds.

### 2.10 Statistical Analysis

Statistical analysis was performed using SPSS 20.0 software. Kolmogorov-Smirnov and Shapiro-Wilk tests were used for data normality tests (**Table S2**). For data with normal distribution, Student’s t-test and one-way analysis of variance (ANOVA) were applied for two-group and multiple-group comparisons, respectively. For data with non-normal distribution, Mann-Whitney U test was applied for comparison of two independent groups, Spearman correlation test was applied for continuous data. Multiple linear regression model for severity associated factors analysis was established first based on all clinical features and DAP content (**Table S3**), and the obtained significant variables were included in a new model to determine the contribution of DAP on severity (**Table S4**). Receiver operating characteristic (ROC) curve was used to evaluate the diagnostic capacity of DAP for severity, besides, age and gender were included for analyses adjustment. *p*<0.05 was considered statistically significant.

## 3 Results

### 3.1 Changes in Histopathology, Enzymology and Gut Microbiota Structure in the SAP Rat Model

**Figure 1:**
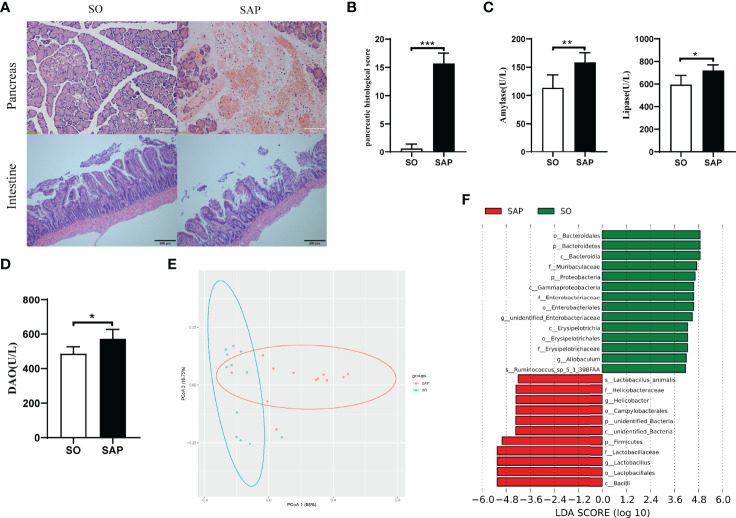
Changes in histopathology, enzymology and gut microbiota structure in the SAP rat model. **(A)** Histopathological observation of the pancreas (HE, ×200) and intestine (HE, ×100) of SO and SAP rats; **(B)** Pancreatic histological score of SO and SAP rats; **(C)** Amylase and lipase levels in the serum of SO and SAP rats; **(D)** Diamine oxidase (DAO) levels in the serum of SO and SAP rats; **(E)** PCoA of the gut microbiota structure and abundance in SO and SAP rats; **(F)** Linear discriminant analysis (LDA) effect size of the SO and SAP groups. SO, sham operation group; SAP, severe acute pancreatitis group. Student’s t-test for Panels **B–D** (n = 6 in each group); *p < 0.5, **p < 0.01, ***p < 0.001.

To evaluate the establishment of the SAP rat model, the histopathology of the pancreas and the levels of serum lipase and amylase were examined. Pancreatic histopathology revealed obvious vacuolization, hemorrhage, necrosis, and infiltration of inflammatory cells in the SAP model compared with SO (**Figure 1A**). The pancreatic pathological scores of the SAP group were higher than those of the SO group (*p*<0.01, **Figure 1B**). Serum amylase and lipase levels were significantly higher in the SAP group than in the SO group (*p*<0.05, **Figure 1C**). The above results suggested that the SAP rat model was successfully prepared. Intestinal histopathology displayed shortened sparse villi with irregular arrangement in the SAP group compared to the SO group, which had dense microvilli arranged in an orderly manner (**Figure 1A**). The serum DAO level was higher in the SAP group than in the SO group, which indicated that the intestinal permeability of SAP rats was increased (**Figure 1D**). In addition, 16S rDNA high-throughput sequencing was employed to identify the changes in the gut microbiota structure and abundance in SAP rats. The sequencing results showed that the sodium taurocholate-induced SAP model altered the bacterial structure of the rat intestine, as evaluated by PCoA (**Figure 1E**). We observed a significantly increased abundance of Firmicutes (phylum), Helicobacteriaceae (family), Helicobacter (genera), and Lactobacillus (genera) and a decreased abundance of Bacteroidetes (phylum), Proteobacteria (phylum), Erysipelotrichaceae (order) and Allobaculum (genera) in SAP rats compared to the SO group (**Figure 1F**). The above results illustrated that the structure and abundance of the gut microflora in SAP rats was changed significantly.

### 3.2 DAP Activates the NOD1/RIP2 Inflammatory Signaling Pathway and Participates in the Systemic Inflammatory Response in SAP Rats

**Figure 2:**
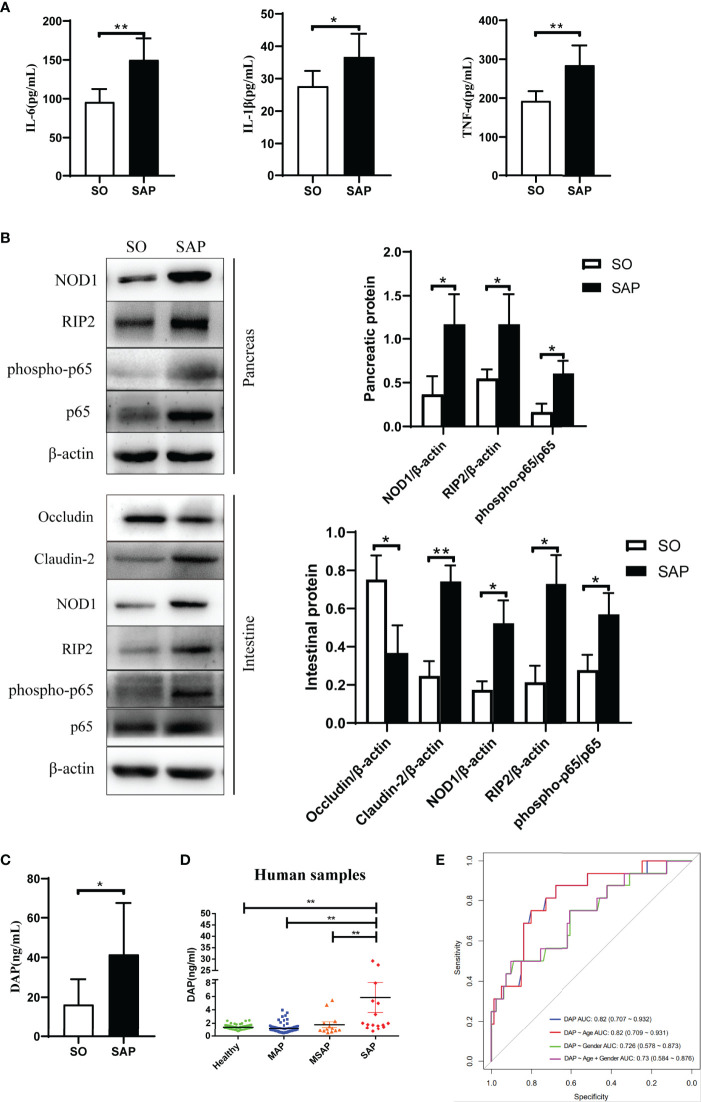
DAP activated the NOD1/RIP2 inflammatory signaling pathway and participated in the systemic inflammatory response in SAP rats, and an elevated serum DAP level was detected in SAP patients. **(A)** The levels of IL-6, IL-1β and TNF-α in the serum of SO and SAP rats; **(B)** Protein expression of pancreatic and intestinal NOD1 signaling pathway, and intestinal barriers proteins Occludin and Claudin-2 by western blotting; **(C)** Determination of serum DAP levels in SO and SAP rats by UHPLC-TOMS; **(D)** Determination of serum DAP levels in patients with AP of different severities; **(E)** Receiver operator characteristic (ROC) analysis for distinguishing between MAP/MSAP and SAP by the serum DAP level. MAP, mild acute pancreatitis; MSAP, moderately severe acute pancreatitis; SAP, severe acute pancreatitis. Student’s t-test for A-C (n = 6 in each group of Panel **A**; n = 3 in each group of Panel **B**; for Panel **C**, n = 8 in SO group and n = 7 in SAP). ANOVA test for Panel **D** and ROC analysis for Panel **E** (n = 56 in healthy group, n = 68 in MAP group, n = 13 in MSAP group, n = 16 in SAP group); *p < 0.5, **p < 0.01.

The NOD1 signaling pathway can be activated by disordered gut microbiota and can recruit downstream RIP2 to promote the inflammatory response. Here, NOD1 and its downstream effectors were evaluated in the intestine and pancreas of SAP rats induced by sodium taurocholate. Serum levels of inflammatory factors were tested as shown in **Figure 2A**. The levels of TNF-α (p<0.01), IL-6 (p<0.01) and IL-1β (p<0.05) in the SAP group were significantly higher than those in the SO group. The western blotting results showed that the expression level of NOD1 and its downstream messenger RIP2 as well as the phosphorylation level of transcription factor NF-κB significantly increased in the SAP group compared with the SO group in both the pancreas and intestine (**Figure 2B**, p<0.05). In addition, the expression of intestinal tight junction proteins changed with a decrease in Occludin and an increase in Claudin-2 (**Figure 2B**), suggesting disruption of the intact intestinal mucosal barrier. Therefore, we wondered whether DAP, as a specific ligand of NOD1, a component of the bacterial cell wall, could be detected in the serum of SAP rats. Based on the UHPLC-TQMS (Waters, USA) platform, we used a derivatization method to detect the serum DAP levels of rats in the two groups. The results showed that the serum DAP level of SAP rats was significantly higher than that of the SO group (p<0.05, **Figure 2C**). These results indicated that the NOD1/RIP2 signaling pathway activated by DAP played a central role in the systemic inflammatory response of SAP rats.

### 3.3 Serum DAP Level of SAP Patients Increased at the Early Stage

We evaluated the changes in serum DAP levels in AP patients and whether they were related to the severity of AP. The serum samples of 153 subjects including 97 AP patients with different severities and 56 healthy individuals were collected upon admission. We utilized the UHPLC-TQMS platform to establish the quantitative detection method of DAP and to detect the serum DAP levels of all subjects. First, Correlation analyses suggested that severity was the only impacting factor of serum DAP content, among 14 clinical features (p<0.001, **Table S2**). The DAP level of SAP patients was obviously higher than that in the other groups (p<0.01, **Figure 2D**). However, there was no significant difference among MAP, MSAP patients and HCs (**Figure 2D**). Multivariate regression analysis based on all clinical features indicated that serum cholesterol (CHOL) and DAP acted as the risk factors of SAP (p<0.001, **Table S3**). Further analysis based on CHOL, DAP and their interaction CHOL:DAP suggested that DAP (p<0.01) was an independent risk factor of SAP, and its interaction with CHOL (p<0.05) also contributed to SAP (**Table S4**). Next, we evaluated the serum DAP level to distinguish between MAP/MSAP and SAP, which is difficult for clinician to evaluate within 48 hours according to existing guidelines. The area under the curve (AUC) was 0.82 (95% CI 0.707 to 0.932), and it still reached an AUC value of 0.73 (95% CI 0.584 to 0.876, **Figure 2E**) after adjustment by including age and gender factors. Therefore, DAP has the potential to be a biomarker as an early indicator of SAP, implying that the gut microbiota is disturbed and that bacterial components enter the blood. This is of great significance for developing therapeutic strategies for SAP at the early stage.

### 3.4 QYKL and Neomycin Regulated Gut Microbiota and Inhibited NOD1 Signaling Pathway to Relieve SAP

#### 3.4.1 QYKL and Neomycin Intervention Improved Pancreatic Damage and Enzymatic Indices in SAP Rats

**Figure 3:**
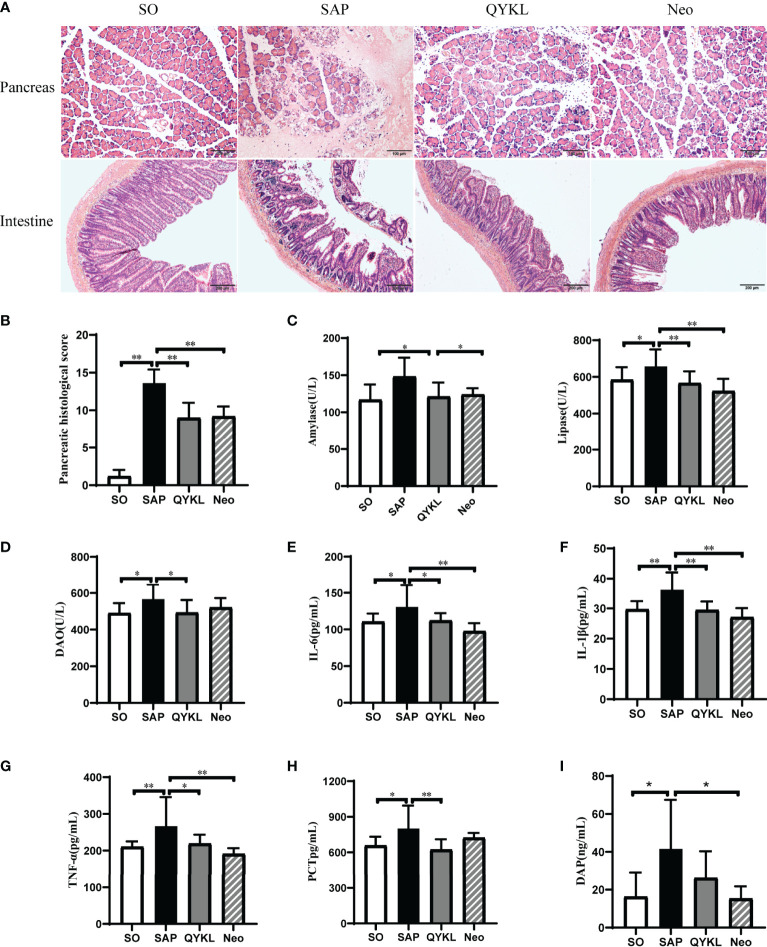
Both Qingyi Keli and neomycin alleviated pancreatic injuries, serum enzymatic indices, intestinal damage and reduced plasmatic inflammatory factors in SAP rats. **(A)** Histological observation of the pancreas (HE, ×200) and intestine (HE, ×100) among the four groups; **(B)** Pancreatic histological scoring among the four groups; **(C)** Amylase and lipase levels in the serum among the four groups. **(D)** DAO levels in serum among the four groups; **(E–H)** IL-6, IL-1β, TNF-α and PCT levels in the serum among the four groups; **(I)** Determination of serum DAP levels in different animal model groups. QYKL, Qingyi Keli treatment group; Neo, neomycin pretreatment group. ANOVA test (n = 5 in each group of Panel **B**, n ≥ 7 in each group of Panel **C–H**, n ≥ 7 in each group of Panel **I**); *p < 0.5, **p < 0.01.

The mortality of rats was 40% in SAP group, 18.8% in QYKL group and 16.7% in Neo group respectively. The present results showed that both QYKL and neomycin intervention could ameliorate the symptoms of SAP rats. Pancreatic histopathology showed slight edema of pancreatic acinar cells, and less inflammatory cell infiltration was observed in the QYKL and Neo groups than in the SAP group (**Figure 3A**). Therefore, the pancreatic pathological scores of the QYKL and Neo groups decreased obviously (p<0.01, **Figure 3B**). Compared with that of the SAP group, the level of amylase was decreased in the QYKL group (p<0.05, **Figure 3C**), as was that in the Neo group, but the difference was not significant. Lipase levels decreased significantly in both the QYKL and Neo groups (p<0.01, **Figure 3C**). The results suggested that both QYKL and neomycin intervention could alleviate pancreatic damage and reduce serum levels of amylase and lipase in the SAP model.

#### 3.4.2 QYKL and Neomycin Intervention Ameliorated SAP-Associated Intestinal Injury and Reduced Systemic Inflammation in SAP Rats

Intestinal histopathology revealed damaged intestinal villi in the SAP group. The QYKL group showed a relatively intact structure and dense intestinal villi with a regular arrangement. Rats in the Neo group showed a better intestinal morphology than those in the SAP group, but the villi were still incomplete and loose relative to those in the SO group (**Figure 3A**). The serum DAO level was significantly decreased in the QYKL group compared to the SAP group (p<0.05, **Figure 3D**), as was that in the Neo group, but the difference was not significant (**Figure 3D**). Serum levels of inflammatory factors were measured and are shown in **Figures 3E–H**. Compared with the SAP group, the levels of TNF-α, IL-6, IL-1β and PCT were decreased significantly in the QYKL group (p<0.05), while the levels of these inflammatory factors, except PCT, were decreased more obviously in the Neo group than in the QYKL group. Importantly, we observed that the serum level of DAP was decreased in both the QYKL- and Neo-treated groups compared with the SAP group according to UHPLC-TQMS detection (**Figure 3I**). These results suggested that both QYKL and Neo could inhibit systemic inflammation in SAP rats.

#### 3.4.3 Disrupted Gut Microbiota Structure in the SAP Group Was Restored in the QYKL Group

**Figure 4:**
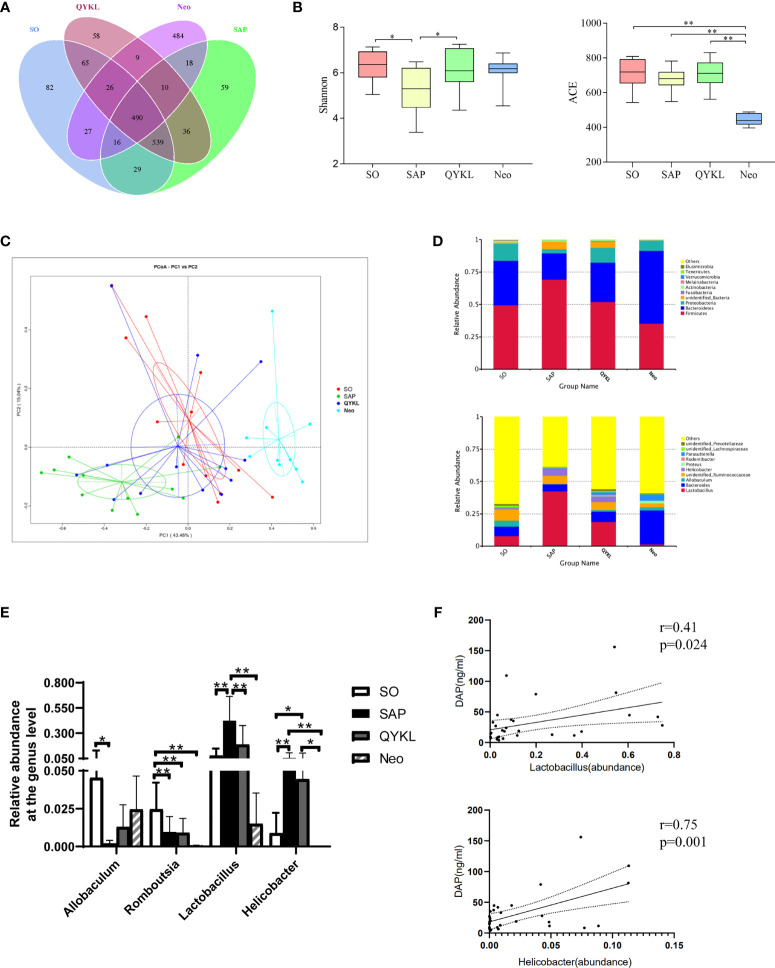
Differences in the gut microbiota components among the four groups. **(A)** OTU numbers among the four groups; **(B)** Alpha diversity reflected by the Shannon index (*p* = 0.04) and ACE index (*p* < 0.01); **(C)** Beta diversity reflected by principal coordinate analysis (PCoA, Adonis analysis, *p* = 0.003); **(D)** Relative abundance of the gut microbiota at the phylum and genus levels; **(E)** Four bacteria that changed significantly in at least two groups. **(F)** Pearson correlation analyses between serum DAP level and the abundance of Lactobacillus and Helicobacter (n = 28). Tukey test for Panel **B**, PCoA for Panel **C**, and ANOVA test for Panel **E** (n = 12 in SO group, n = 12 in SAP group, n = 13 in QYKL group, n = 10 in Neo group); *p < 0.5, **p < 0.01.

To explore the discrepancy in the gut microbiota composition among the SO group (n=12), SAP group (n=12), QYKL group (n=13) and Neo group (n=10), 47 fecal samples were examined using 16S rRNA gene sequencing. A total of 3,119,296 high-quality sequence reads were generated, with 66,368 reads on average for each sample. All sequences were clustered into 2023 OTUs based on a 97% nucleotide similarity cutoff for species-level bacterial phylotypes. Common and unique OTUs among the four groups are shown in **Figure 4A**. The Shannon and ACE indices reflected the gut microbiota diversity and abundance, respectively. Compared with the SO group, the SAP group had a significantly lower Shannon index, which was restored in the QYKL group (Tukey test, p=0.04, **Figure 4B**). The species abundance was not significantly different among the SO, SAP and QYKL groups but was decreased dramatically in the Neo group (p < 0.01, **Figure 4B**). The β diversity reflected by PCoA based on the weighted UniFrac distances revealed that the overall microbial composition of the SAP and Neo groups deviated from that of the SO group in two different ways (Adonis analysis, p=0.003, **Figure 4C**), while the microbiota structure in the QYKL group was very close to the distribution of the SO group (**Figure 4C**). The histograms in **Figure 4D** show the species abundance among different groups. At the phylum level, compared with the SO group, a larger proportion of Firmicutes and a lower proportion of Bacteroidetes and Proteobacteria were present in the SAP group. Compared with levels in the SAP group, the proportion of Firmicutes decreased, and the proportions of Bacteroidetes and Proteobacteria increased in both the QYKL and Neo groups. At the genus level, four bacteria, including Allobaculum, Romboutsia, Lactobacillus and Helicobacter, exhibited apparent abundance differences in at least two groups (**Figure 4E**). Univariate statistical analysis indicated that Allobaculum was significantly decreased in the SAP group compared with the SO group, while its abundance was increased in the QYKL and Neo groups. Romboutsia in the SO group was significantly more abundant than that in the other three groups (p<0.01, **Figure 4E**); Lactobacillus and Helicobacter were higher in the SAP than in the SO group, and its abundance was decreased in the QYKL and Neo groups, especially in the Neo group (p<0.01, **Figure 4E**). Both of Lactobacillus (Huang et al., 2017) and Helicobacter (Kim et al., 2015) contain DAP component, and showed positive correlation with serum DAP level of rats (**Figure 4F**). The above results illustrated that the gut microflora was obviously disordered in the SAP models and tended to recover under QYKL treatment. The regulatory effect of QYKL was mild and bidirectional, while Neo could directly eliminate sensitive bacteria.

#### 3.4.4 Both QYKL and Neomycin Intervention Can Inhibit the Activation of the NOD1 Signaling Pathway

**Figure 5:**
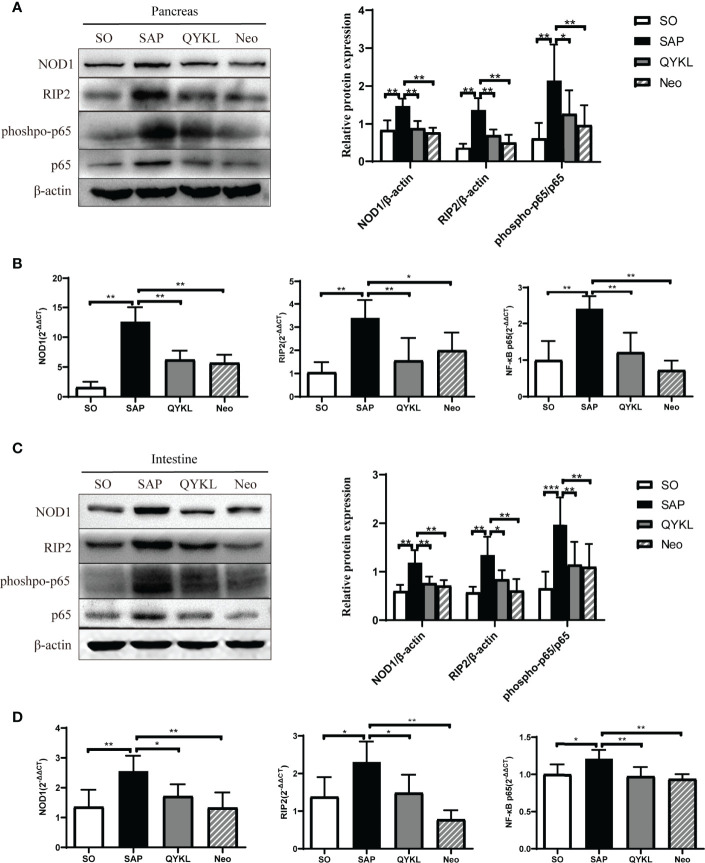
Expression of the NOD1 signaling pathway in the pancreas and intestine. **(A)** Expression of NOD1, RIP2, phospho-p65 and p65 in the pancreas by western blotting; **(B)** Comparison of the expression levels of each mRNA of NOD1 signaling pathway in the pancreas among the four groups; **(C)** Expression of NOD1, RIP2, phospho-p65 and p65 in the intestine by western blotting; **(D)** Comparison of the expression levels of each mRNA of NOD1 signaling pathway in the intestine among the four groups. ANOVA test (n = 6 in each group); *p < 0.5, **p < 0.01, ***p < 0.001.

Activation of the NOD1 signaling pathway plays a crucial role in cerulein-induced mouse pancreatitis (Tsuji et al., 2012). Here, NOD1 and its downstream effectors were evaluated in the intestine and pancreas of each group. Compared with levels in the SAP group, the expression levels of NOD1, RIP2 and the phosphorylation level of NF-κB significantly decreased in the QYKL and neomycin groups at both the protein and mRNA levels in the pancreas (p<0.05, **Figures 5A, B**) and intestine (p < 0.05, **Figures 5C, D**). The results indicated that QYKL and neomycin treatments depressed the activation of the NOD1-RIP2 signaling pathway at both the protein and mRNA levels in SAP rats. Notably, the trend of the changes in DAP levels (**Figure 3I**) was consistent with the expression of the NOD1-RIP2 signaling pathway. These results suggested that the upregulation of NOD1-RIP2 in SAP rats might be due to the increase in serum DAP levels, which can be reduced by QYKL and neomycin treatment. Therefore, the ameliorative effect of intervention with QYKL and neomycin for SAP might be related to regulating the gut microbiota-DAP-NOD1/RIP2 signaling axis.

## 4 Discussion

Recently, accumulating evidence has indicated that bacteria in the gut participate in AP progression, although the mechanism underlying the gut-pancreas crosstalk is unclear. Herein, we reported that the activation of intestinal and pancreatic NOD1-RIP2-NF-κB probably depended on the DAP component from intestinal bacteria entering into the circulation, which may be associated with exacerbation of AP. Additionally, restoration of the gut microbiota was observed during recovery from SAP, accompanied by attenuation of intestinal and pancreatic NOD1 pathway activation and a decrease in DAP abundance in serum, as demonstrated in the QYKL treatment group and the neomycin pretreatment group. Notably, the clinical results showed that the serum DAP level of SAP patients increased at the early stage, which indicated that DAP had the potential to become a biomarker as an early signal of SAP.

In this study, SAP model rats were successfully established as evaluated by pancreatic pathological changes and enzymatic indices. After SAP induction, intestinal tissue showed obvious morphologic alterations and disorganized villus arrangement. Research has suggested that the main causes leading to intestinal damage include fluid loss-induced hypovolemia, splanchnic vasoconstriction and ischemia–reperfusion injury (Capurso et al., 2012). DAO secreted by epithelial cells is usually regarded as a permeability indicator of the intestine (Banks et al., 2013), and increased DAO levels in the plasma of SAP rats suggests the occurrence of intestinal barrier dysfunction following structural disruption, which drives extensive inflammatory reactions (de Jong et al., 2016; Werge et al., 2016). The elevation of TNF-α, IL-6, IL-1β and PCT in SAP rats verified the development of systemic inflammation. After QYKL and Neo intervention, injuries in both the pancreas and intestine were alleviated, and plasmatic inflammatory factors were reduced, suggesting that QYKL and Neo could effectively inhibit the inflammatory response. Interestingly, DAO and PCT were only significantly reduced after QYKL treatment but not with Neo treatment; the former reflected gut integrity, and the latter was closely related to bacterial infection (Schuetz et al., 2017). These results suggested that QYKL might have a better protective role in intestinal barrier function than Neo, which might be due to antibiotic-associated dysbiosis (Soares et al., 2017). The protective effect of QYKL was consistent with previous reports that Qingyi decoction had therapeutic effects in SAP-induced intestinal barrier dysfunction (Zhang et al., 2015; Su et al., 2019).

Oral administration is the most common method of administering medicine in traditional Chinese medicine, and QYKL is no exception, as it has extensive effects on the intestinal microflora. Oral administration of herbal medicine to the gastrointestinal tract can modulate the colonization predominance of gut microbiota by altering the intestinal milieu; conversely, microbiota may metabolite herbs into a variety of active ingredients, and cometabolism of the host and microbiota is recognized as an important mechanism for herbal efficacy (Jiang et al., 2016). In this study, 16S rDNA sequencing showed that gut microbiota diversity was significantly decreased and that the microbiota structure was notably altered in SAP rats, which was consistent with other studies (Banks et al., 2013; Tan et al., 2015; Chen et al., 2017). Treatment with QYKL improved microbiota diversity and rehabilitated the disturbed gut microbiota to nearly normal levels. Neo potently inhibited gut microbiota abundance and greatly influenced its structure, but it was far different from normal. The restoration of intestinal microflora by QYKL was milder and closer to normal than that of Neo. Additionally, we observed that Allobaculum and Romboutsia were reduced in SAP rats. Previous studies have shown that Allobaculum was a beneficial species (Baldwin et al., 2016), and Romboutsia abundance was inversely correlated with TNF-α, IL-1β, D-lactic and DAO (Zhu et al., 2018); hence, the intestinal environment affected by SAP might restrain potential anti-inflammatory probiotics. Lactobacillus and Helicobacter increased in SAP rats, and the former was reversed after QYKL treatment. Lactobacillus is traditionally recognized as a beneficial species that ferments polysaccharides into organic acids, while it appears to play a detrimental role in SAP rats. Similar results were reported by Zhu et al., who found that Lactobacillales was positively correlated with TNF-α, IL-1β and DAO levels in patient serum (Zhu et al., 2018), suggesting that the enrichment of these bacteria might correlate with the production of inflammatory factors and increased intestinal permeability, but the specific causality is still obscure. Furthermore, Lancet reported that prophylactic probiotics predominantly composed of Lactobacillus may increase mortality risk in patients with predicted SAP (Besselink et al., 2008). Therefore, we speculated that Lactobacillus might not play a protective role in AP progression, which needs further study to reveal its concrete mechanism. Another bacterium that increased in SAP was Helicobacter, and studies have shown that intragastric Helicobacter species, such as H. pylori, were closely associated with peptic ulcer disease, nonulcer dyspepsia and gastric cancer (Crowe, 2019). Enterohepatic Helicobacter species are closely related to hepatobiliary diseases (Smet and Menard, 2020). After QYKL treatment, the abundance of the two kinds of bacteria decreased, while Neo pretreatment almost eliminated both Lactobacillus and Helicobacter in SAP rats. Neo affects mostly the gastrointestinal tract because of its low absorptivity (Sasseville, 2010) and can inhibit bacteria bearing DAP, such as Lactobacillus (Huang et al., 2017) and Helicobacter (Kim et al., 2015), so it was adopted in our study as a positive reference to investigate the effect of QYKL on DAP-containing bacteria.

**Figure 6:**
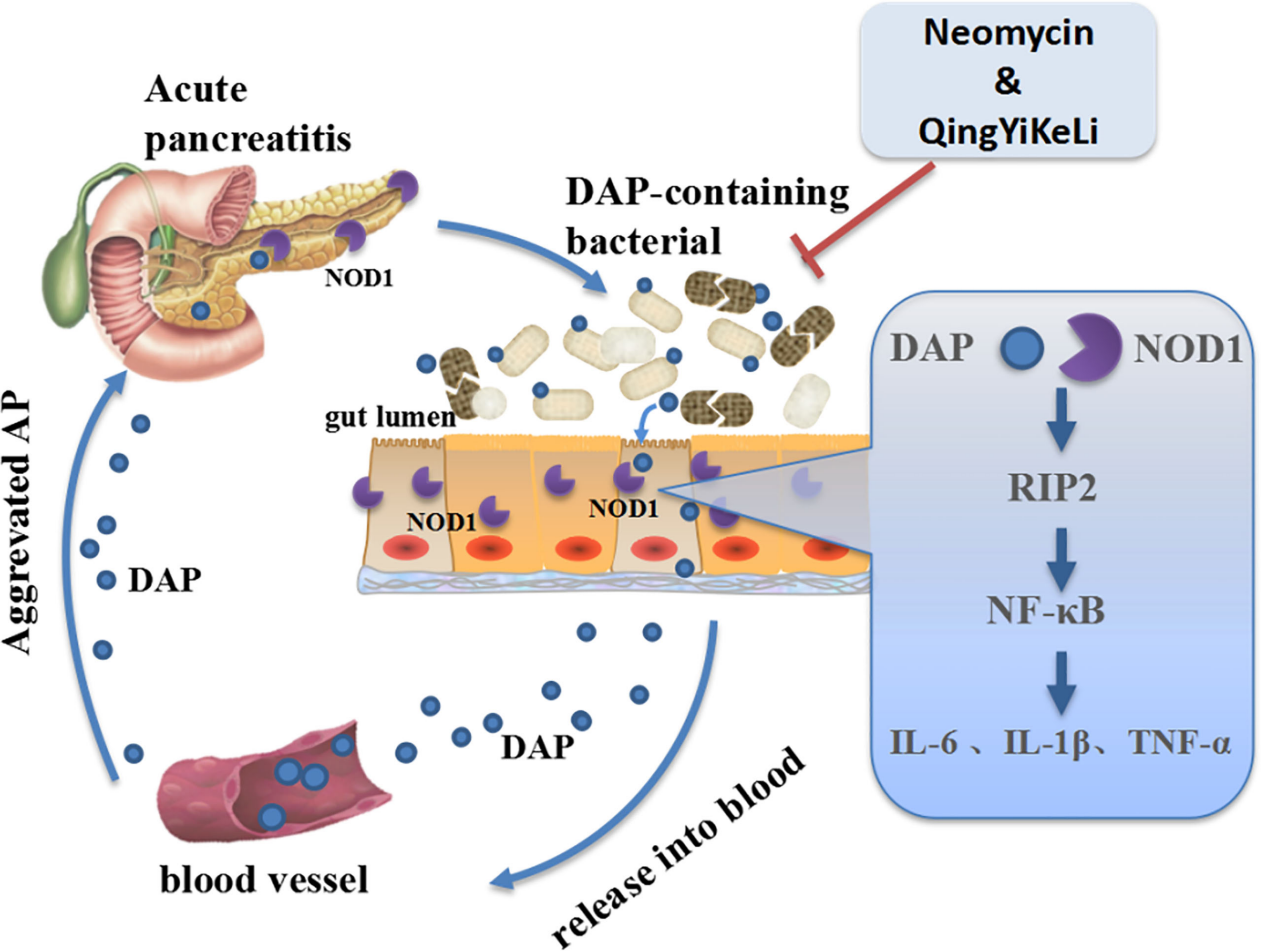
Schematic illustration showing that QYKL and neomycin ameliorate severe acute pancreatitis through the gut microbiota-DAP/NOD1 signaling axis.

The molecular mechanisms underlying the exacerbation of pancreatitis caused by gut microbiota dysbiosis are not well understood. In this study, we observed synchronous changes in gut microbiota dysbiosis, increases in serum DAP levels and activation of intestinal and pancreatic NOD1/RIP2/NF-κB during SAP. However, with treatment with QYKL and Neo, which is an antibiotic that DAP-containing bacteria are sensitive to, gut microbiota restoration was accompanied by a decrease in the serum DAP content and a weakened NOD1/RIP2/NF-κB signaling pathway. Previous work has demonstrated that NOD1 responses were the key factors in the development of pancreatitis, as reflected in the experimental pancreatitis caused by cholecystokinin hyperstimulation (Tsuji et al., 2012; Watanabe et al., 2017). Although NOD2, a homologous molecule of NOD1 and capable of activating RIP2, was reported to participate in the intestinal injury of SAP (Xu et al., 2018), it did not induce a comparable pancreas-located injury, neither an elevation of serum amylase and lipase as NOD1, when co-stimulated with low-dose cholecystokinin receptor agonist (Tsuji et al., 2012). Furthermore, our results showed that the increased expression of NOD1 and its adapter RIP2 was followed by the upregulation of the phosphorylated NF-κB transcription factor. NF-κB activation can induce IL-6 synthesis, both the increased IL-6 and NOD1-mediated another cytokine type I interferon are able to activate STAT3, by which the activation of NF-κB and STAT3 synergize to promote a maximal production of chemokine MCP-1 (Tsuji et al., 2012). This create an inflammatory feedback circuit to facilitate the production of inflammatory factors and destroy intestinal tight junction proteins and cause intestinal epithelial barrier dysfunction (Shawki and McCole, 2017). Once the intestinal barrier breaks down, DAP, as a small molecule, has a larger probability than the whole bacterium of entering the circulation and translocating into distant organs. This might explain the study by Tsuji et al. (2012), who found that the gut microbiota mediated cerulein-induced pancreatitis through activation of pancreatic acinar NOD1 to a much greater extent than TLR4. Activation of TLR4 by gram-negative bacteria derived LPS can also induce downstream NF-κB and MAPK signaling through myeloid differentiation protein-88 (MyD88), and was reported to aggravate AP though damaging intestinal barrier (Zheng et al., 2019). In other words, the interaction between enteral microbiota and the distant pancreas highly depends on the DAP component, and NOD1 and TLR4 can cooperate to activate NF-κB signaling in SAP. These results suggested that gut microbiota dysbiosis might exacerbate inflammation through the activation of the NOD1 signaling pathway by DAP. Notably, QYKL and Neo intervention restrained overactivated NOD1 signaling and thus reduced the production of inflammatory factors. The gene expression profile based on a gene chip assay indicated that the effects of Qingyi decoction on SAP rats were closely related to NOD-like receptors (Zhu et al., 2014). Collectively, our results indicated that in SAP, the gut microbiota was disordered, DAP-containing bacteria increased, and DAP was released into the blood system, which activated the NOD1-RIP2 signaling pathway, followed by aggravation of pancreatitis. The alleviation of SAP by QYKL and Neo might be associated with restoring gut microflora disturbance, lowering the serum DAP level and repressing the expression of the NOD1-RIP2 signaling pathway (**Figure 6**).

DAP is a ligand for NOD1 that can induce both innate and adaptive immune responses (Uehara et al., 2006; Guzelj and Jakopin, 2020). Upon DAP recognition by NOD1, an immune inflammatory response is generated *via* NF-κB and mitogen-associated protein kinases (MAPKs) (Chamaillard et al., 2003), while DAP has also been implicated in other innate immunity mechanisms, such as apoptosis (Inohara and Nunez, 2003) and autophagy (Travassos et al., 2010), as well as cancer metastasis (Jiang et al., 2020). With regard to the detection of DAP, recent studies mostly adopted fluorescent reporter cells to measure NOD1 responsiveness and provided relative quantification information about NOD1 ligands, thus lacking direct evidence that the NOD1 ligand DAP exists in serum. In this study, based on the UHPLC-TQMS platform, we established a quantitative detection method of serum DAP for the first time by using the precolumn derivatization method and isotope-labeled internal standard. Animal experiments showed that the serum DAP level of SAP rats increased significantly and decreased after QYKL treatment or neomycin intervention. A limitation of our study lays in not considering gender impact on gut microbiota or serum DAP level of rat, but our analyses based on clinical samples showed that there was no significant difference in serum DAP level between male and female AP patients or healthy volunteers. Moreover, elevated serum DAP levels at the early stage were verified in SAP patients, while in our clinical samples, they did not change significantly among MAP and MSAP patients compared with healthy subjects, which is worthy of further study in a larger sample size. Overall, this detection method can accurately and quantitatively analyze serum DAP levels. More importantly, early detection of SAP is of great significance for developing therapeutic strategies and improving the prognosis of SAP.

## 5 Conclusion

In summary, our work highlights the role of DAP in the microbiota-host interplay, which has a significant impact on AP progression. We revealed that the gut microbiota-DAP-NOD1/RIP2 signaling axis participated in the progression of AP. Our results verified that the serum DAP contents could be an indicator of the incidence of SAP at an early stage. Moreover, we demonstrated that oral administration of the traditional Chinese medicine QYKL could ameliorate SAP by regulating the gut microbiota-pancreatic axis. Our work provides a new potential early warning signal and target of treatment of SAP.

## Data Availability Statement

The datasets presented in this study can be found in online repositories. The names of the repository/repositories and accession number(s) can be found below: CNGB Sequence Archive(CNSA) of China National GeneBank DataBase (CNGBdb) with accession number CNP0002549.

## Ethics Statement

The studies involving human participants were reviewed and approved by the Ethics Committee of the First Affiliated Hospital of Dalian Medical University (YJ-KS-KY-2019-93). The patients/participants provided their written informed consent to participate in this study. The animal study was reviewed and approved by the Ethics Committee of Animal Experiments of Dalian Medical University. Written informed consent was obtained from the individual(s) for the publication of any potentially identifiable images or data included in this article.

## Author Contributions

DS, PY, JJ, AND JL designed the study protocol and managed the study. JJ, JL, and QL conducted the experimental work. GZ, QZ, BQ, and LZ collected healthy and patient samples, and provided clinical and scientific expertise to this project. JJ, CP, and FL conducted the data analysis. JJ, JL, PY, and DS wrote and edited the paper. All authors contributed to the article and approved the submitted version.

## Funding

This work was supported by the National Natural Science Foundation of China (no.81873156,82004152), National Key Research and Development Plan of China (2018YFE0195200), 2018 TCM clinical subjects Capacity building project of Health and Family Planning Commission in Liaoning Province (194-2018125), Liaoning Provincial Natural Science Foundation of China (2019-MS-082).

## Conflict of Interest

The authors declare that the research was conducted in the absence of any commercial or financial relationships that could be construed as a potential conflict of interest.

## Publisher’s Note

All claims expressed in this article are solely those of the authors and do not necessarily represent those of their affiliated organizations, or those of the publisher, the editors and the reviewers. Any product that may be evaluated in this article, or claim that may be made by its manufacturer, is not guaranteed or endorsed by the publisher.
