# Supplementary figures and images for: Gut Microbiota-Derived Diaminopimelic Acid Promotes the NOD1/RIP2 Signaling Pathway and Plays a Key Role in the Progression of Severe Acute Pancreatitis
Source: Front Cell Infect Microbiol. 2022 Jun 22;12:838340. doi: 10.3389/fcimb.2022.838340 (PMC9257083; doi:10.3389/fcimb.2022.838340)

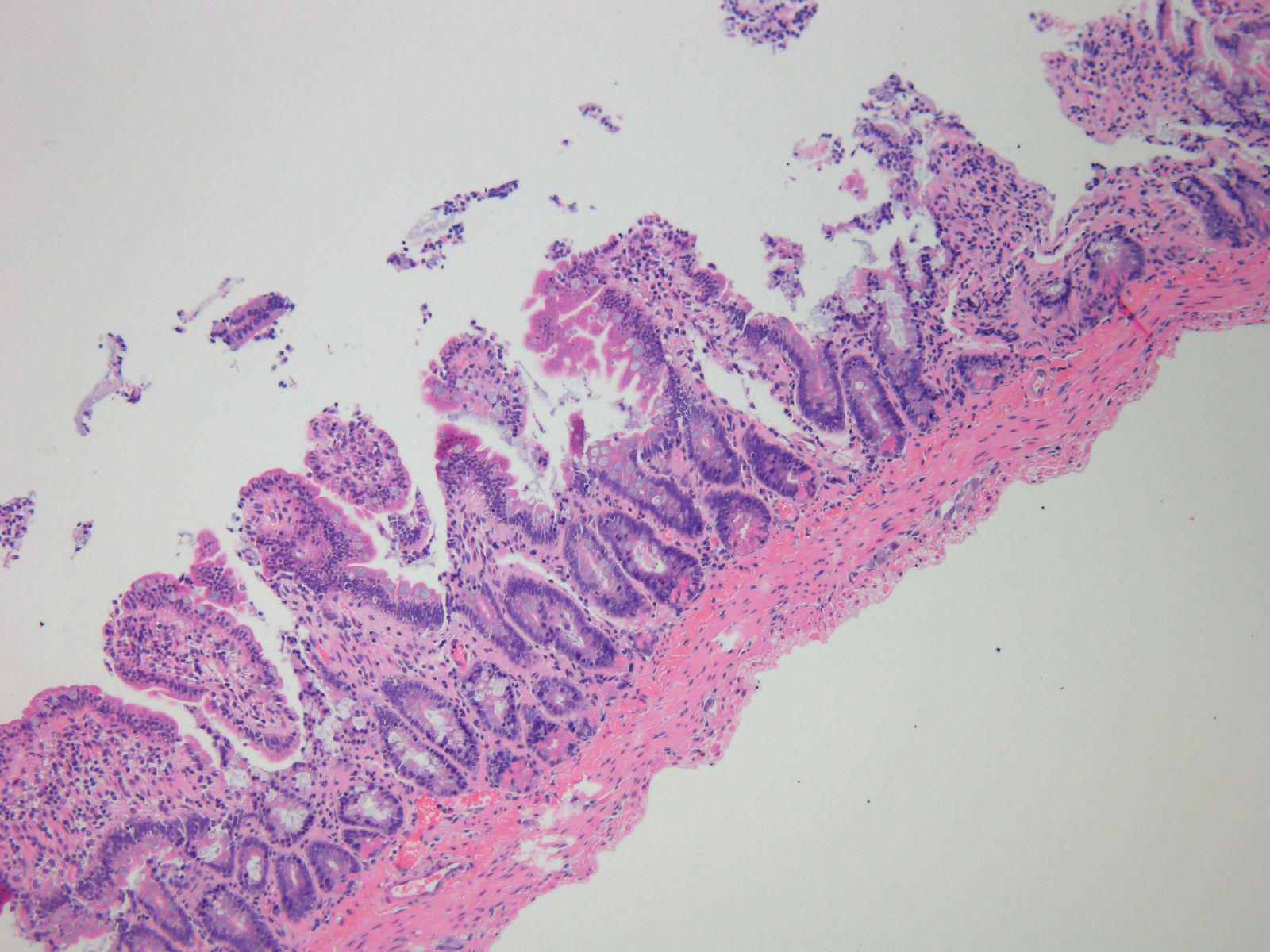

Supplement: Supplementary file 1 [file Image_1.tif]

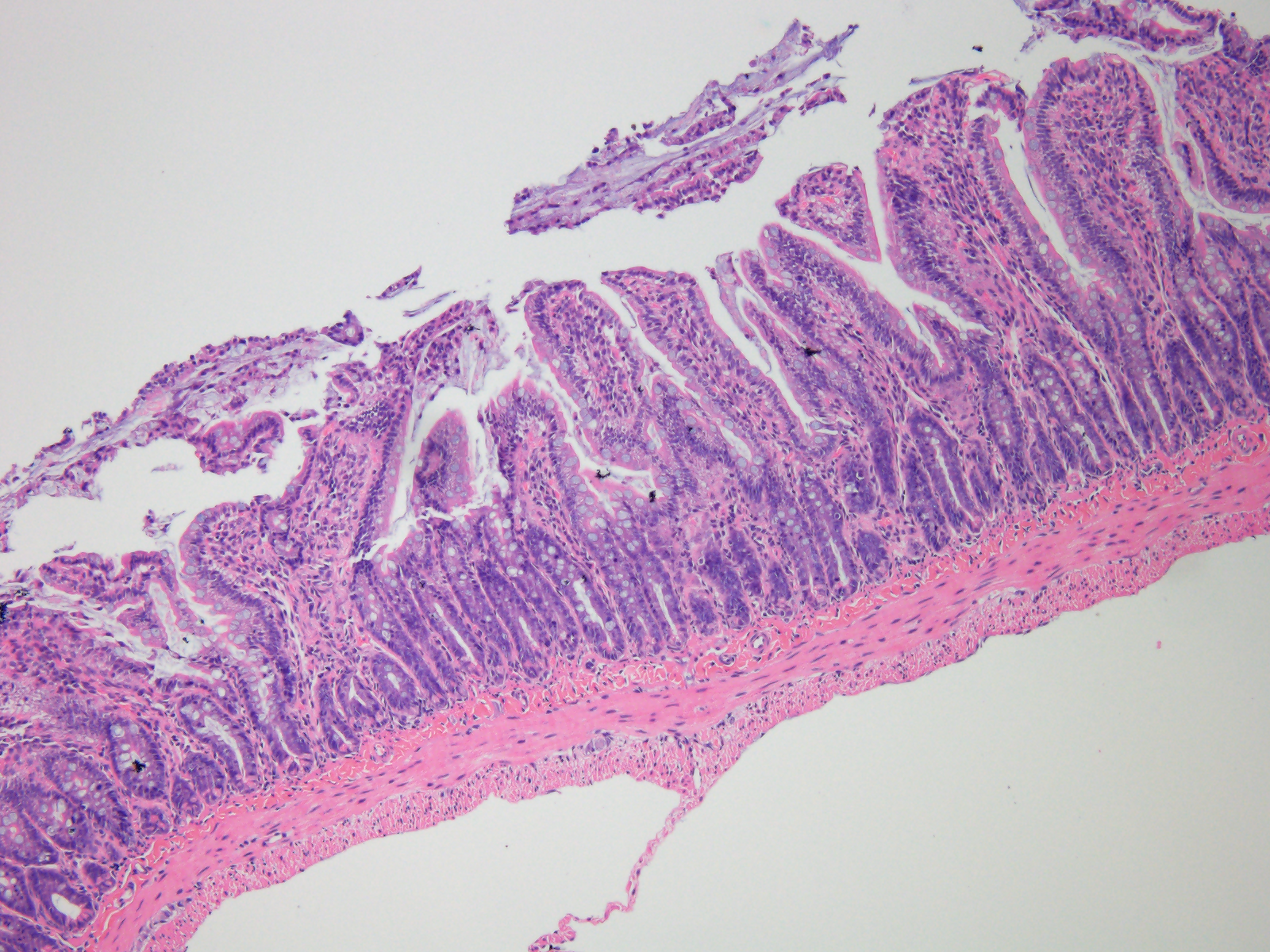

Supplement: Supplementary file 2 [file Image_2.tif]

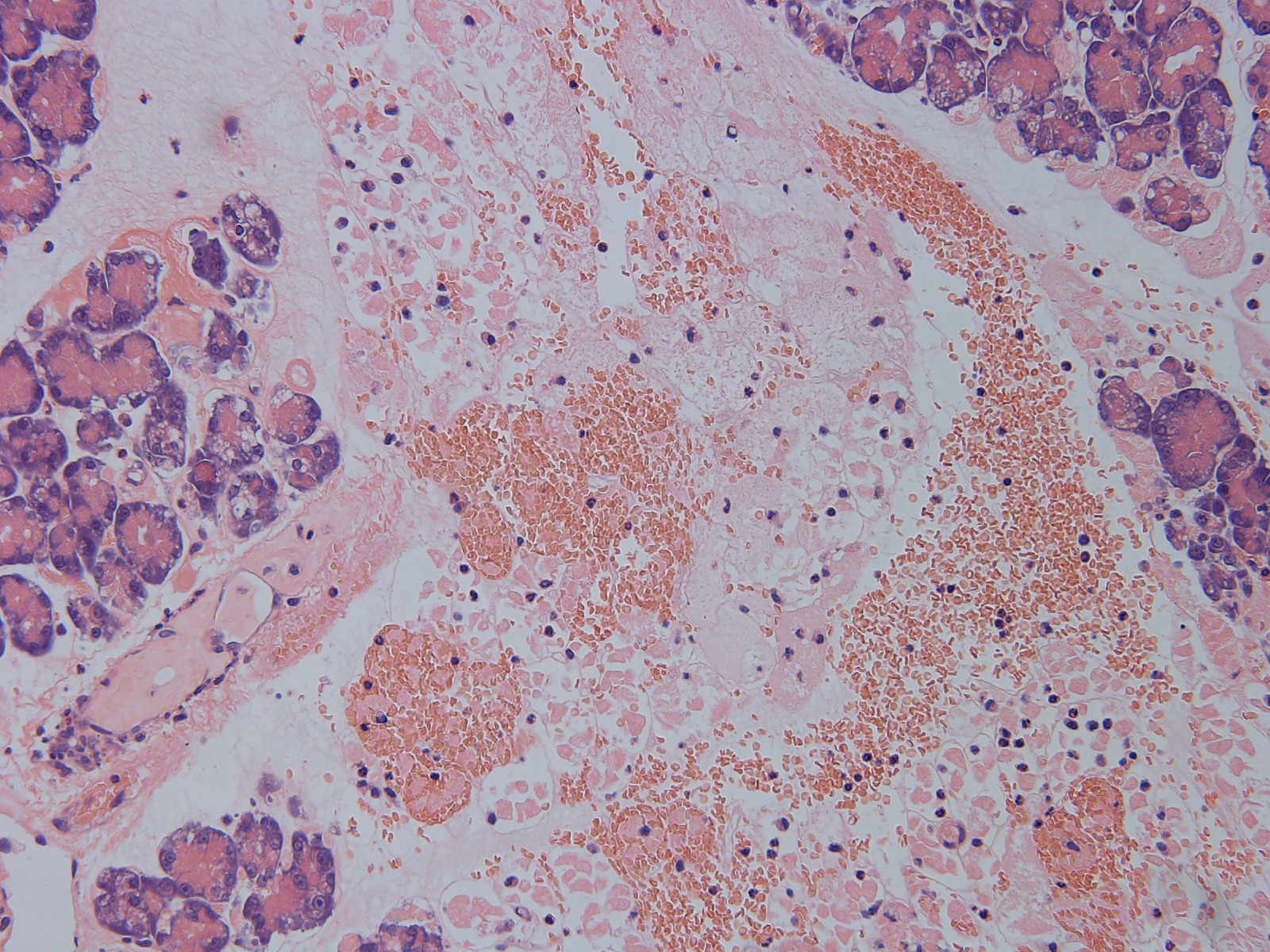

Supplement: Supplementary file 3 [file Image_3.tif]

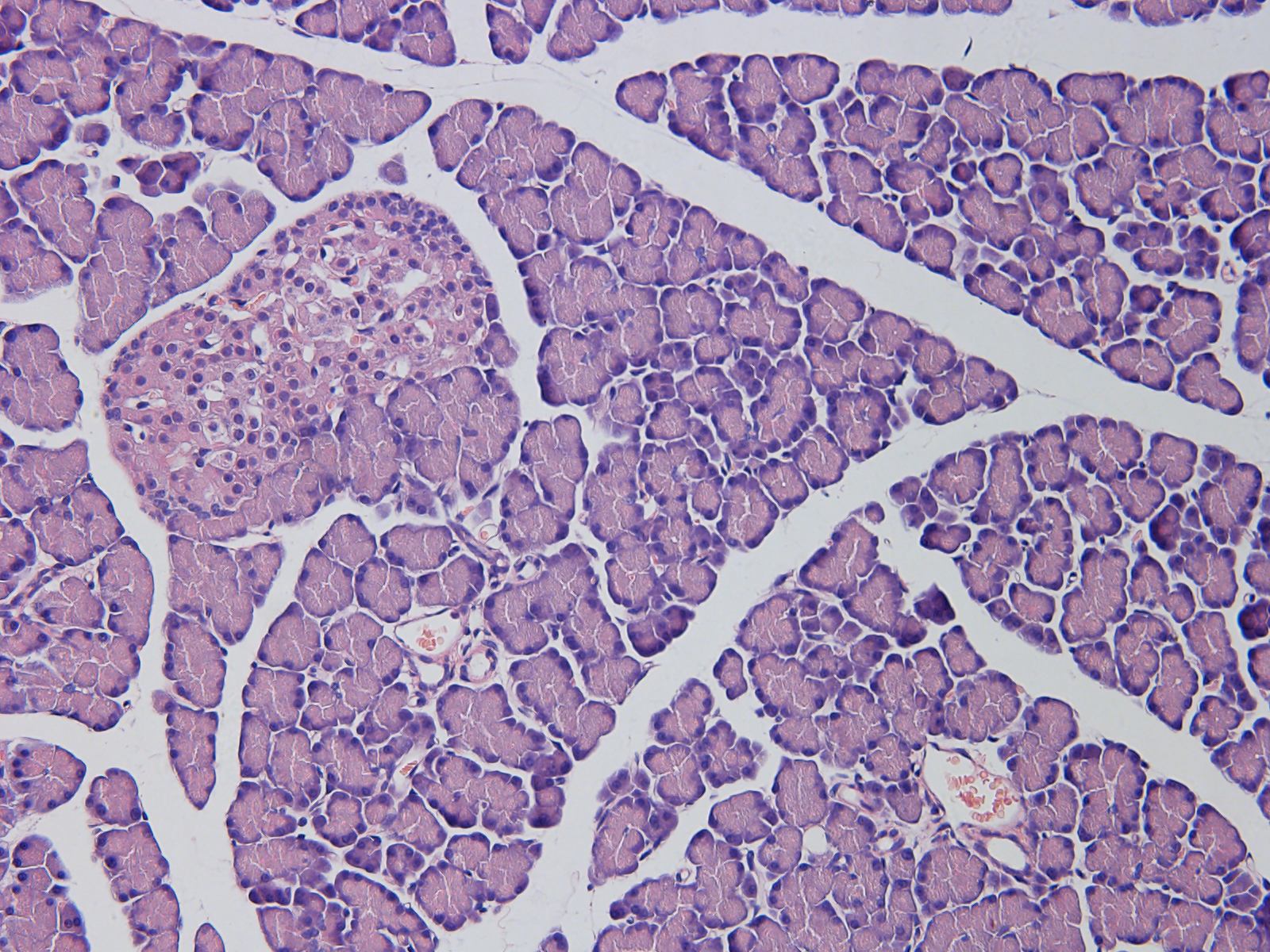

Supplement: Supplementary file 4 [file Image_4.tif]

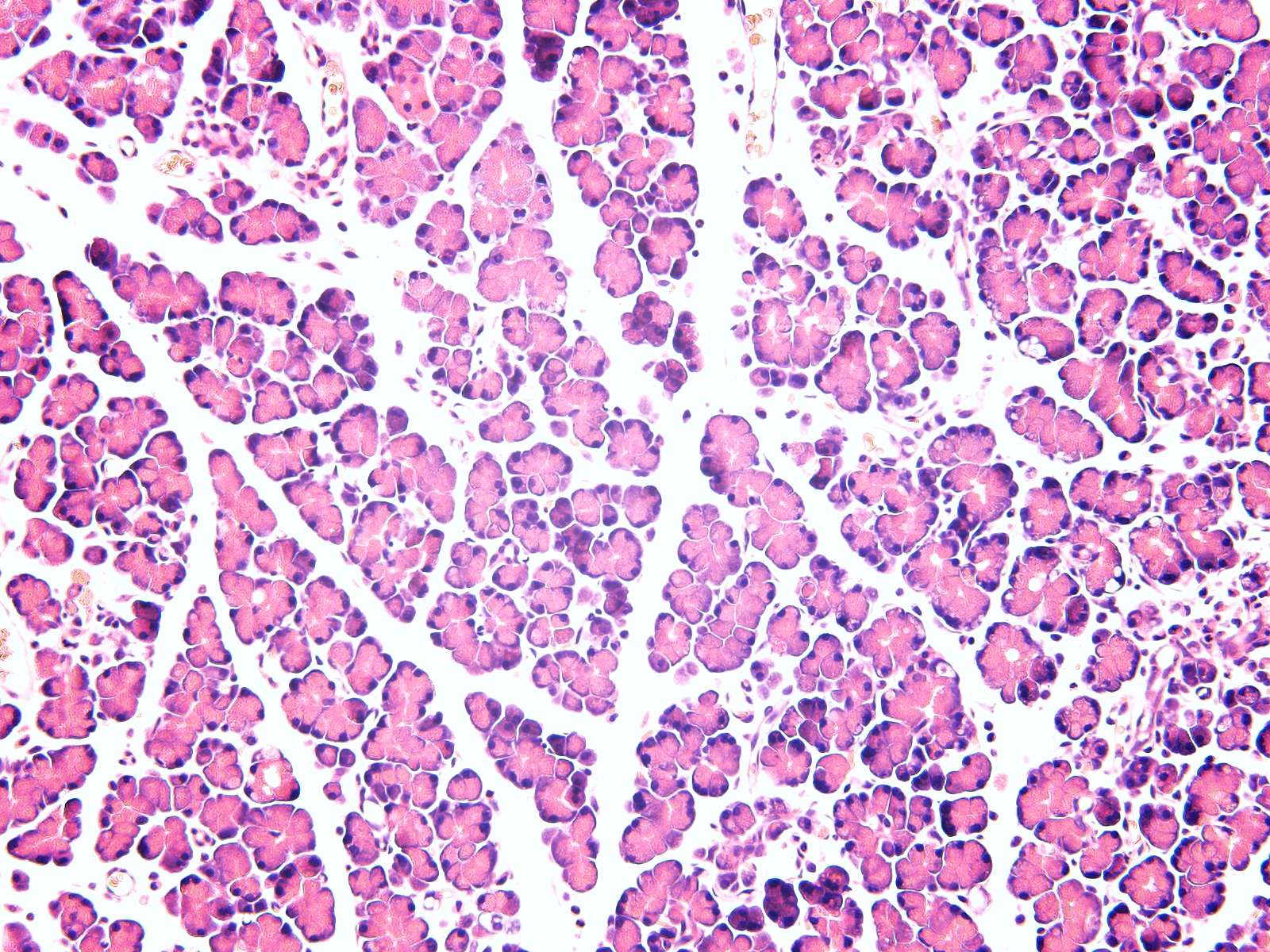

Supplement: Supplementary file 5 [file Image_5.tif]

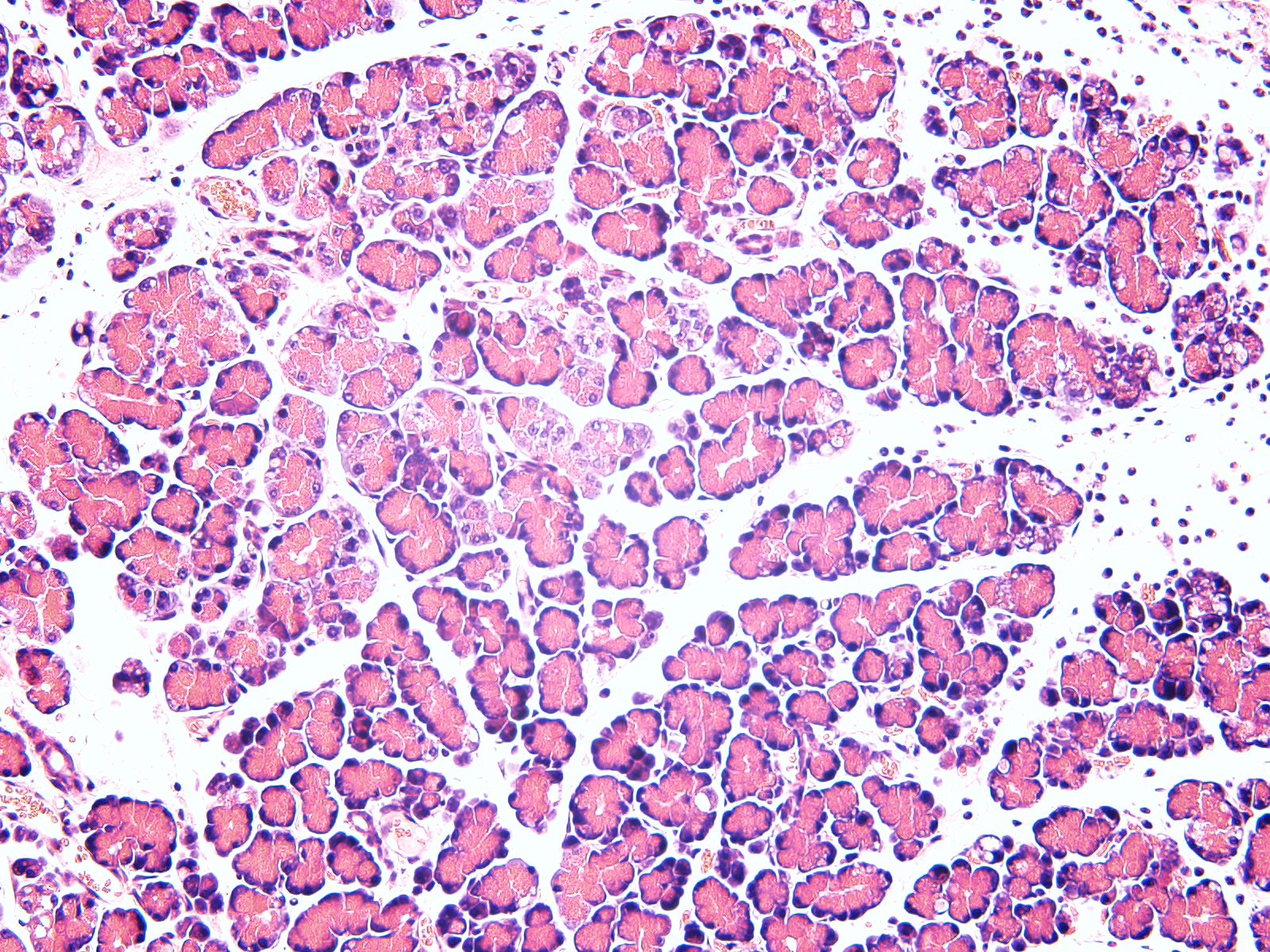

Supplement: Supplementary file 6 [file Image_6.tif]

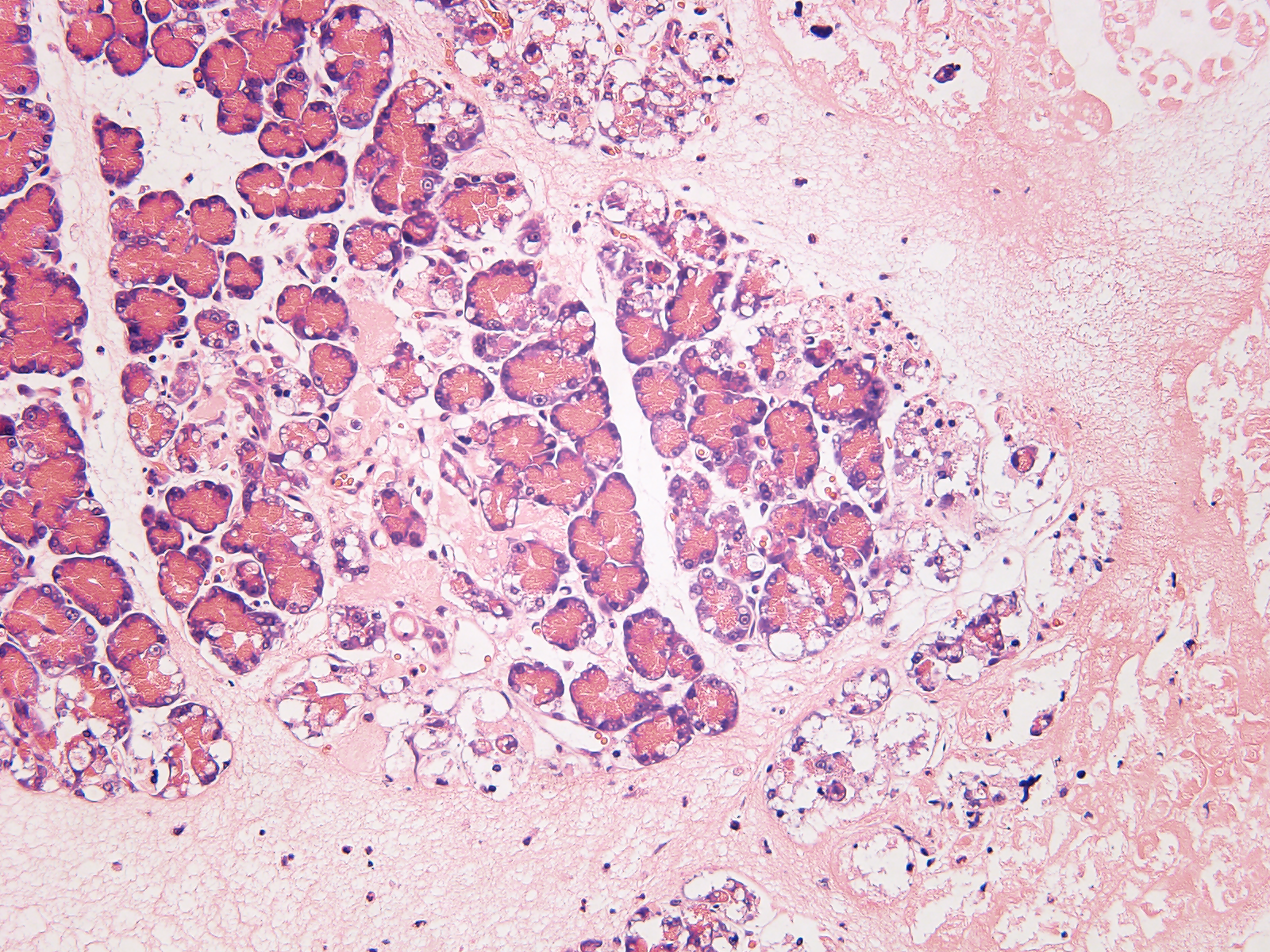

Supplement: Supplementary file 7 [file Image_7.tif]

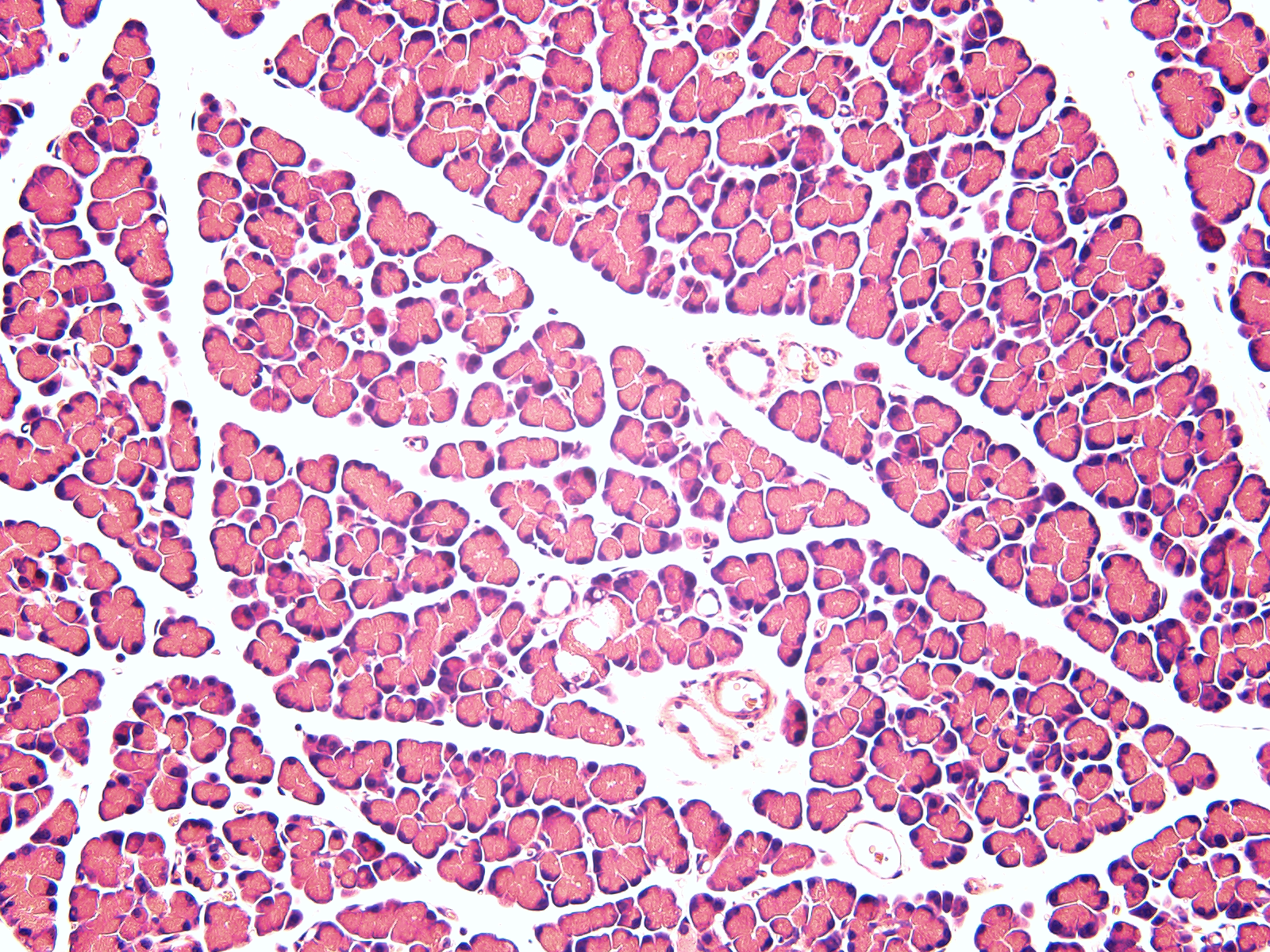

Supplement: Supplementary file 8 [file Image_8.tif]

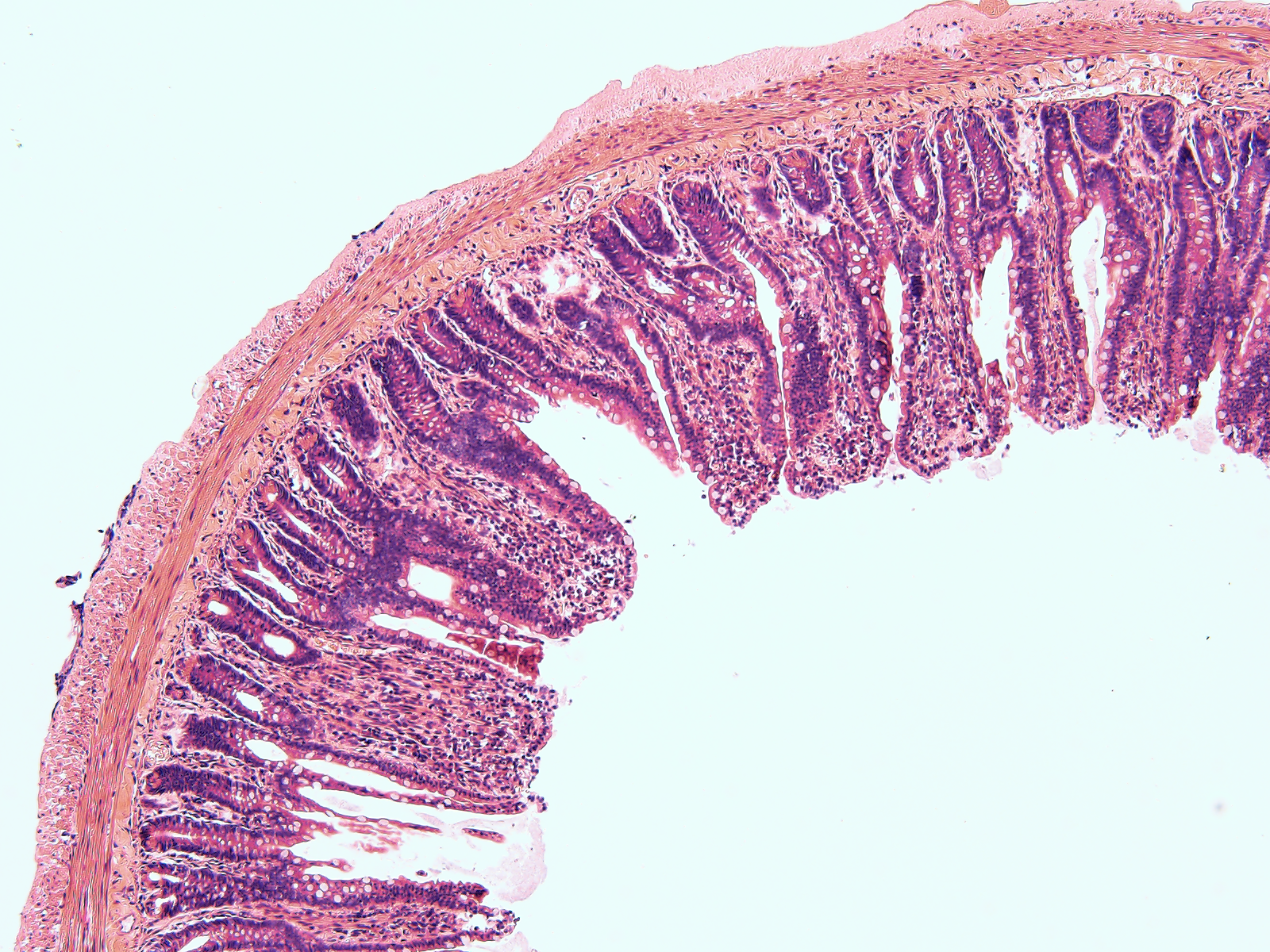

Supplement: Supplementary file 9 [file Image_9.tif]

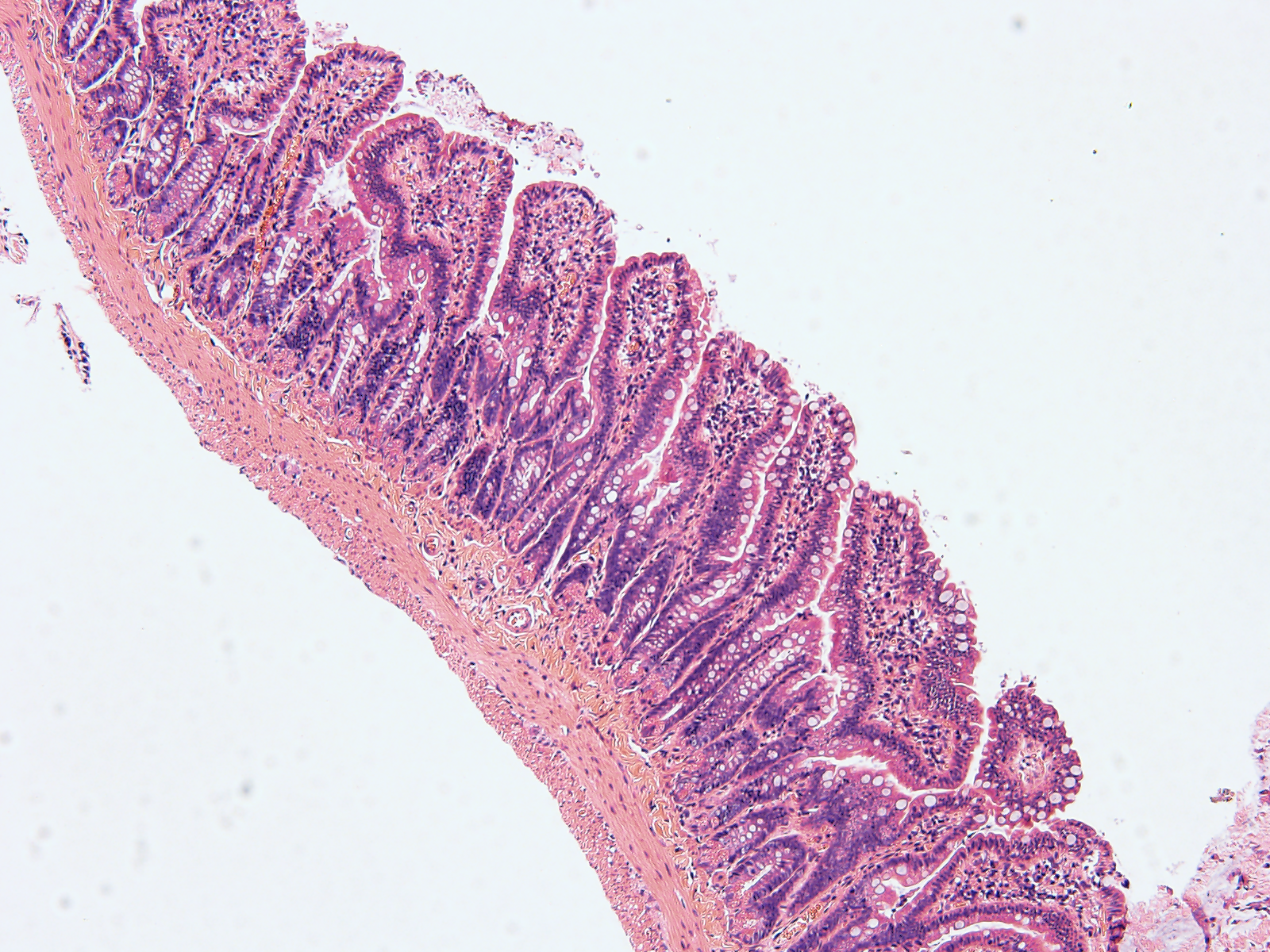

Supplement: Supplementary file 10 [file Image_10.tif]

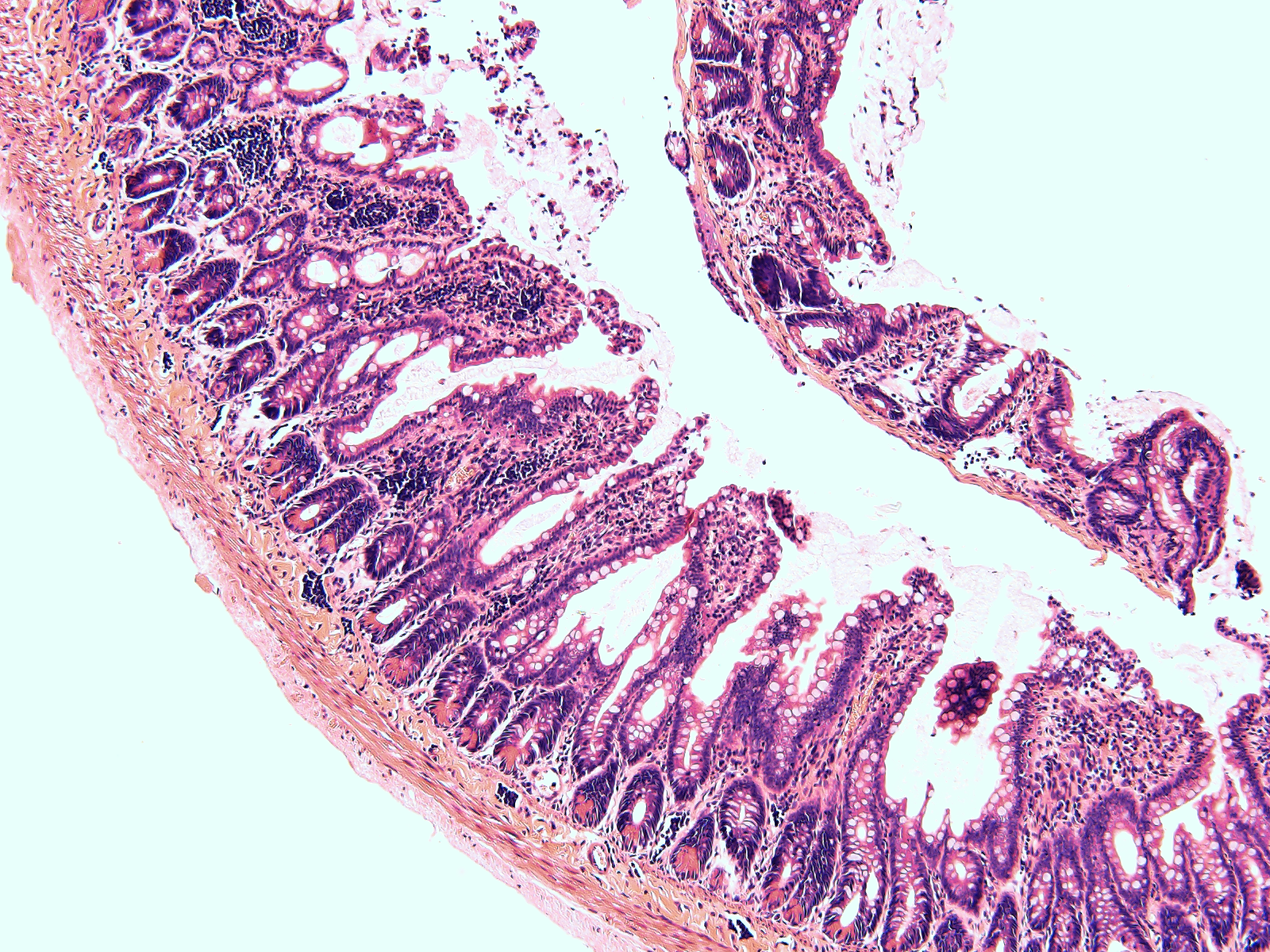

Supplement: Supplementary file 11 [file Image_11.tif]

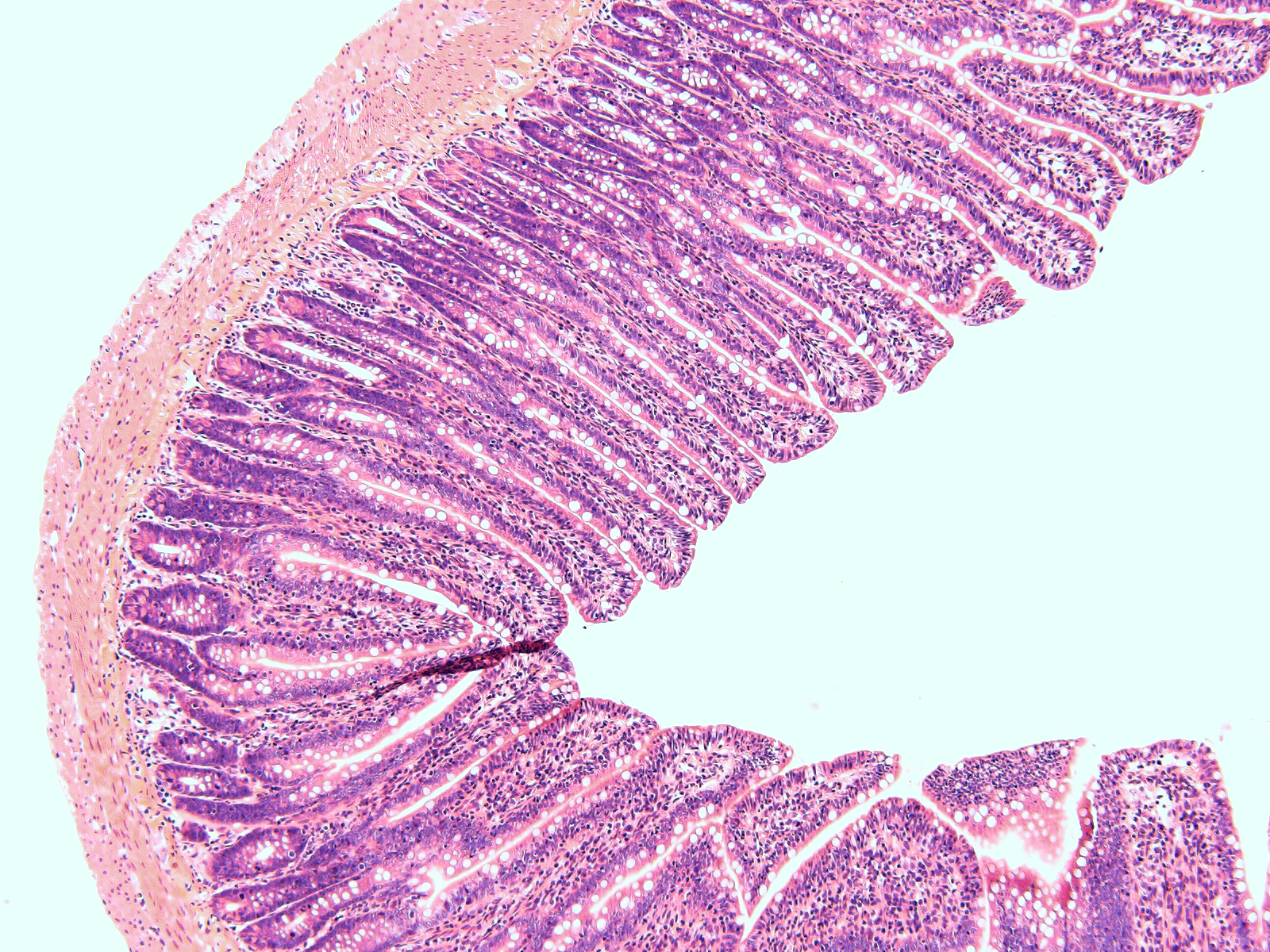

Supplement: Supplementary file 12 [file Image_12.tif]
