# Supplementary material for: Gut Microbiota-Derived Diaminopimelic Acid Promotes the NOD1/RIP2 Signaling Pathway and Plays a Key Role in the Progression of Severe Acute Pancreatitis
Source: Front Cell Infect Microbiol. 2022 Jun 22;12:838340. doi: 10.3389/fcimb.2022.838340 (PMC9257083; doi:10.3389/fcimb.2022.838340)
Supplement: Supplementary file 13 [file Table_1.docx]

Supplementary Material

**Table S1**. Clinical features of the recruited subjects

|  | AP (n=97) | | | | HC (n=56) | |
| --- | --- | --- | --- | --- | --- | --- |
|  | MAP (n=68) | MSAP (n=13) | SAP (n=16) | *P* value | HC (n=56) | *P* value |
| Gender, F/M | 22/46 | 4/9 | 3/13 |  | 17/39 |  |
| Age, years | 51 ± 14 | 51 ± 19 | 44 ± 17 | > 0.05 | 50 ± 7 | > 0.05 |
| ALT, U/L | 336.8 ± 373.7 | 230.7 ± 373.6 | 110.6 ± 96.6 | 0.094 | 30.1 ± 30.7 | <0.001 |
| AST, U/L | 265.3 ± 266.2 | 127.0 ± 163.5 | 110.6 ±96.6 | 0.055 | 23.4 ± 12.0 | <0.001 |
| γ-GT, U/L | 321.5 ± 292.7 | 195.0 ± 139.5 | 204.5±197.3 | > 0.05 | 30.4 ± 22.5 | <0.001 |
| ALP, U/L | 172.9 ± 95.3 | 124.3 ± 87.9 | 94.2 ± 48.1 | < 0.05 | 73.4 ± 20.9 | <0.001 |
| CHOL, mmol/L | 6.9 ± 2.5 | 3.3 ± 2.2 | 3.4±1.0 | < 0.05 | 5.3 ± 1.1 | > 0.05 |
| TG, mmol/L | 6.8 ± 5.6 | 5.3 ± 7.6 | 3.1±2.5 | > 0.05 | 1.4 ± 1.0 | <0.001 |
| GLU, mmol/L | 8.8 ± 2.9 | 13.4 ± 9.5 | 9.7±4.4 | < 0.05 | 5.1 ± 0.7 | <0.001 |
| WBC, 10^9/L | 13.3 ± 3.4 | 14.3 ± 5.9 | 14.7±7.7 | > 0.05 | 5.7 ± 1.7 | <0.001 |
| %NEUT | 78.6 ± 17.0 | 89.5 ± 4.9 | 77.9±22.7 | > 0.05 | 57.0 ± 6.5 | <0.001 |
| Serum amylase, U/dL | 1109.2 ± 1315.3 | 892.8±926.5 | 814.6±724.9 | > 0.05 | - |  |
| Serum lipase, U/dL | 4915.0 ± 4991.7 | 2987.4±3349.2 | 3202.4±3055.4 | > 0.05 | - |  |

**Table S2**. Correlation analysis between DAP and clinical features.

|  | Normality test | | |  | | Correlation analysis with DAP | | | |
| --- | --- | --- | --- | --- | --- | --- | --- | --- | --- |
|  | statistics | Degree of freedom | Significance | |  | | statistics | significance |  |
| DAP | 0.342 | 97 | 0.000 | |  | | - | - |  |
| Female | 0.690 | 28 | 0.000 | |  | | -1.454 | 0.146 |  |
| Male | 0.341 | 68 | 0.000 | |  | |  |  |  |
| MAP/MSAP | 0.212 | 81 | 0.000 | |  | | -3.961 | 0.000 |  |
| SAP | 0.566 | 16 | 0.000 | |  | |  |  |  |
| Amylase | 0.235 | 90 | 0.000 | |  | | -0.066 | 0.536 |  |
| Age | 0.110 | 97 | 0.006 | |  | | 0.054 | 0.598 |  |
| Lipase | 0.214 | 86 | 0.000 | |  | | -0.096 | 0.379 |  |
| WBC | 0.132 | 77 | 0.002 | |  | | -0.037 | 0.749 |  |
| NEUT | 0.317 | 78 | 0.000 | |  | | -0.040 | 0.727 |  |
| ALT | 0.262 | 62 | 0.000 | |  | | -0.163 | 0.205 |  |
| AST | 0.235 | 64 | 0.000 | |  | | -0.137 | 0.279 |  |
| γGT | 0.245 | 71 | 0.000 | |  | | -0.010 | 0.931 |  |
| ALP | .872 | 43 | 0.000 | |  | | 0.046 | 0.771 |  |
| CHOL | .946 | 37 | 0.071 | |  | | -0.155 | 0.360 |  |
| TG | .777 | 40 | 0.000 | |  | | -0.157 | 0.332 |  |
| Glu | 0.186 | 68 | 0.000 | |  | | 0.096 | 0.437 |  |

Correlation analyses were performed to investigate the relationships between the included clinical features and DAP content. Normality test were conducted by Kolmogorov-Smirnov test for variables with n > 50 and Shapiro-Wilk test for variables with n ≤ 50 respectively. Spearman correlation test (statistics refers to ρ-value) was used to analyze continuous variables with non-normal distribution. Mann-Whitney U test (statistics refers to z-value) was used to analyze dichotomic variables with non-normal distribution.

**Table S3**. Multivariate regression model based on DAP and clinical features for severity associated factors analysis.

| Variable | Estimate | | Std. error | t value | *P*-value |
| --- | --- | --- | --- | --- | --- |
| Gender | 0.02715 | | 0.05021 | 0.541 | 0.589538 |
| Age | -0.00282 | | 0.00191 | -1.476 | 0.142148 |
| SA | 9.766E-07 | | 0.00005309 | 0.018 | 0.985352 |
| SL | -0.000003863 | | 0.0000137 | -0.282 | 0.778303 |
| WBC | 0.01083 | | 0.005552 | 1.951 | 0.053045 |
| NEUT | -0.0007217 | | 0.001598 | -0.452 | 0.652174 |
| ALT | -0.0002853 | | 0.0001691 | -1.687 | 0.093926 |
| AST | 0.0001851 | | 0.0002299 | 0.805 | 0.422165 |
| γ-GT | 0.00009725 | | 0.0001528 | 0.636 | 0.525626 |
| ALP | -0.00004262 | | 0.0005628 | -0.076 | 0.939743 |
| CHOL | -0.06898 | | 0.01857 | -3.715 | 0.000294 |
| TG | 0.008357 | | 0.00899 | 0.93 | 0.354178 |
| Glu | 0.007545 | | 0.005944 | 1.269 | 0.206471 |
| DAP | | 0.03404 | 0.006901 | 4.932 | 0.0000023 |

A linear regression model “lm (SAP ~ Gender +Age +SA +SL +WBC +NEUT +ALT +AST +GT +ALP +CHOL +TG +Glu +DAP” was applied. Severity (MAP/MSAP=0, SAP=1) was set as dependent variable. DAP content and clinical features including gender, age, SA (serum amylase), SL (serum lipase), WBC (white blood cells), NEUT (neutrophils), ALT (alanine aminotransferase), AST (aspartate aminotransferase), γ-GT (gamma-glutamyl transpeptidase), ALP (alkaline phosphatase), CHOL (cholesterol), TG (triglycerides), Glu (glucose) were set as independent variables. Estimate represents the intercept of the regression equation. Std. error represents the standard error of the regression estimate in linear model. T value represents the ratio of the estimate to the standard error. *P*-value represents the significance level of each variable in the model. Multiple R-squared: 0.3396, adjusted R-squared: 0.2726.

**Table S4**. Multivariate regression model based on DAP, CHOL and their interactions for severity associated factors analysis.

| Variable | Estimate | Std. error | t value | *P*-value |
| --- | --- | --- | --- | --- |
| CHOL | -0.01147 | 0.02404 | -0.477 | 0.63413 |
| DAP | 0.16424 | 0.05458 | 3.009 | 0.00308 |
| CHOL:DAP | -0.02829 | 0.01213 | -2.332 | 0.02107 |

Linear regression model based on DAP, CHOL and their interaction CHOL:DAP were established. Severity (MAP/MSAP=0, SAP=1) was set as dependent variable. Variables including CHOL, DAP and CHOL:DAP were set as independent variables. Estimate represents the intercept of the regression equation. Std. error represents the standard error of the regression estimate in linear model. T value represents the ratio of the estimate to the standard error. *P*-value represents the significance level of each variable in the model. Multiple R-squared: 0.2834, adjusted R-squared: 0.2690.
